# LIMCH1-enriched extracellular vesicles promote vascular permeability in early-onset preeclampsia

**DOI:** 10.1126/sciadv.aeb8806

**Published:** 2026-01-28

**Authors:** Seiko Matsuo, Akira Yokoi, Takafumi Ushida, Kosuke Yoshida, Hironori Suzuki, Masami Kitagawa, Eri Asano-Inami, Hiroaki Yamada, Rika Miki, Sho Tano, Kenji Imai, Ichiro Nagata, Shota Kawaguchi, Takao Yasui, Yusuke Yamamoto, Hiroaki Kajiyama, Tomomi Kotani

**Affiliations:** ^1^ Department of Obstetrics and Gynecology, Nagoya University Graduate School of Medicine, Nagoya, Japan.; ^2^ Nagoya University Institute for Advanced Research, Nagoya, Japan.; ^3^ Japan Science and Technology Agency (JST), FOREST, Saitama, Japan.; ^4^ Division of Reproduction and Perinatology, Center for Maternal-Neonatal Care, Nagoya University Hospital, Nagoya, Japan.; ^5^ Laboratory of Integrative Oncology, National Cancer Center Research Institute, Tokyo, Japan.; ^6^ Department of Obstetrics and Gynecology, Kurume University School of Medicine, Kurume, Japan.; ^7^ Maternal Fetal Health Laboratory, Research Institute, Nozaki Tokushukai Hospital, Daito, Osaka, Japan.; ^8^ Bell Research Center for Reproductive Health and Cancer, Nagoya University Graduate School of Medicine, Nagoya, Japan.; ^9^ Department of Obstetrics and Gynecology, Japanese Red Cross Aichi Medical Center Nagoya Daini Hospital, Nagoya, Japan.; ^10^ Shin Nippon Biomedical Laboratories Ltd., Kagoshima, Japan.; ^11^ Department of Life Science and Technology, Institute of Science Tokyo, Nagatsuta 4259, Midori-ku, Yokohama 226-8501, Japan.; ^12^ Research Institute for Quantum and Chemical Innovation, Institutes of Innovation for Future Society, Nagoya University, Furo-cho, Chikusa-ku, Nagoya 464-8603, Japan.; ^13^ Department of Obstetrics and Gynecology, Hamamatsu University School of Medicine, Hamamatsu, Japan.

## Abstract

Preeclampsia (PE) is a major pregnancy complication characterized by hypertension and multiple end-organ dysfunctions; however, its detailed pathogenesis remains unclear. Extracellular vesicles (EVs) play diverse and critical roles in intercellular communication, and we have demonstrated interaction between EVs and vascular endothelial cells. Through serum proteomic analysis, we identified LIM and calponin homology domain–containing protein 1 (LIMCH1) as a PE-associated EV protein that is highly expressed in PE placentas, particularly in syncytiotrophoblasts, which release EVs into the maternal circulation. LIMCH1-enriched EVs (LIMCH1-EVs) increased endothelial permeability in vitro. Transcriptome analysis revealed that LIMCH1-EVs disrupted endothelial cell-cell junction assembly by suppressing the expression of the tight junction protein ZO-1. Furthermore, administration of LIMCH1-EVs promoted pulmonary vascular permeability in vivo. These findings suggest a role of LIMCH1-EVs in EV-associated vascular endothelial dysfunction, a central pathology of PE. In addition, this study provides insights into mechanisms that may contribute to PE-associated pulmonary edema, which have not yet been clarified.

## Teaser

LIMCH1-enriched extracellular vesicles promote endothelial permeability, which is a central pathology of preeclampsia.

## INTRODUCTION

Preeclampsia (PE) is a major complication of pregnancy, affecting 5 to 7% of pregnancies, and is characterized by hypertension and multiple end-organ dysfunctions, including renal failure, liver dysfunction, myocardial injury, eclampsia, pulmonary edema, and, in the most severe cases, maternal death (*1*–*3*). Globally, approximately 75,000 mothers and 500,000 infants die each year from PE (*4*, *5*). Currently, no effective therapy other than iatrogenic delivery has been established, often resulting in preterm birth. Therefore, elucidating the underlying mechanisms and developing effective treatments remain urgent clinical priorities (*6*).

The central pathogenesis of PE, particularly early-onset PE (Eo-PE), is thought to involve abnormal placentation during early pregnancy and systemic endothelial dysfunction in the second and third trimesters. The two-stage theory has been widely accepted as a framework for understanding the pathophysiology of PE (*2*, *7*–*9*). In the first stage, shallow spiral artery remodeling during early pregnancy leads to abnormal placental vasculature and underperfusion. Consequently, apoptosis of placental cells [primarily syncytiotrophoblasts (SCTs)], oxidative stress, endoplasmic reticulum stress, and mitochondrial dysfunction resulting from abnormal placentation contribute to the increased release of proinflammatory cytokines, reactive oxygen species, anti-angiogenic factors, and extracellular vesicles (EVs) into the maternal circulation. In the second stage, these circulating factors induce systemic endothelial dysfunction, leading to various clinical manifestations (*5*). Although angiogenic imbalance [e.g., soluble fms-like tyrosine kinase-1 (sFlt-1) and placental growth factor] has been proposed as one of the central mechanism in PE, the pathways responsible for the development of diverse end-organ dysfunctions [e.g., pulmonary edema and posterior reversible encephalopathy syndrome (PRES)] associated with PE remain unclear (*5*).

EVs are lipid bilayer vesicles released by all living cells that contain proteins and nucleic acids and are responsible for intercellular communication and various signaling pathways (*10*, *11*). Small EVs (sEVs) with diameters of 50 to 200 nm are the most studied EV subtype (*12*). sEVs have been implicated in various pathological conditions, including cancer, autoimmune diseases, and infectious diseases (*13*). SCTs are multinucleated, terminally differentiated cells that cover the entire surface of the placental villi and are reported to be the primary source of placenta-derived EVs (*14*). Recent evidence suggests that SCT-derived EVs are internalized by endothelial cells and drive the endothelial dysfunction in vitro (*15*, *16*). Moreover, administration of sEVs derived from PE serum disrupts the blood-brain barrier (BBB) and administration of placenta-derived EVs causes a PE-like phenotype in mice (*17*, *18*). Therefore, it is plausible that sEVs derived from PE placentas affect the vascular endothelial cells and contribute to PE development (*19*). However, the details of PE-related sEVs and mechanisms that induce PE remain unknown. In addition, specific EVs associated with various end-organ dysfunctions in PE have not yet been identified.

In this study, we focused on the proteins in sEVs derived from Eo-PE serum and performed liquid chromatography–tandem mass spectrometry (LC-MS/MS) to investigate how PE-related EVs contribute to endothelial dysfunction associated with PE. We identified LIM and calponin homology domain–containing protein 1 (LIMCH1) as an Eo-PE–related EV protein and investigated the effects of LIMCH1-enriched EVs (LIMCH1-EVs) on vascular endothelial cells. This study offers valuable insights into the underlying mechanisms of Eo-PE and clarifies the role of LIMCH1-EVs.

## RESULTS

### Identification of LIMCH1 as Eo-PE-associated EV protein

**Fig. 1.**
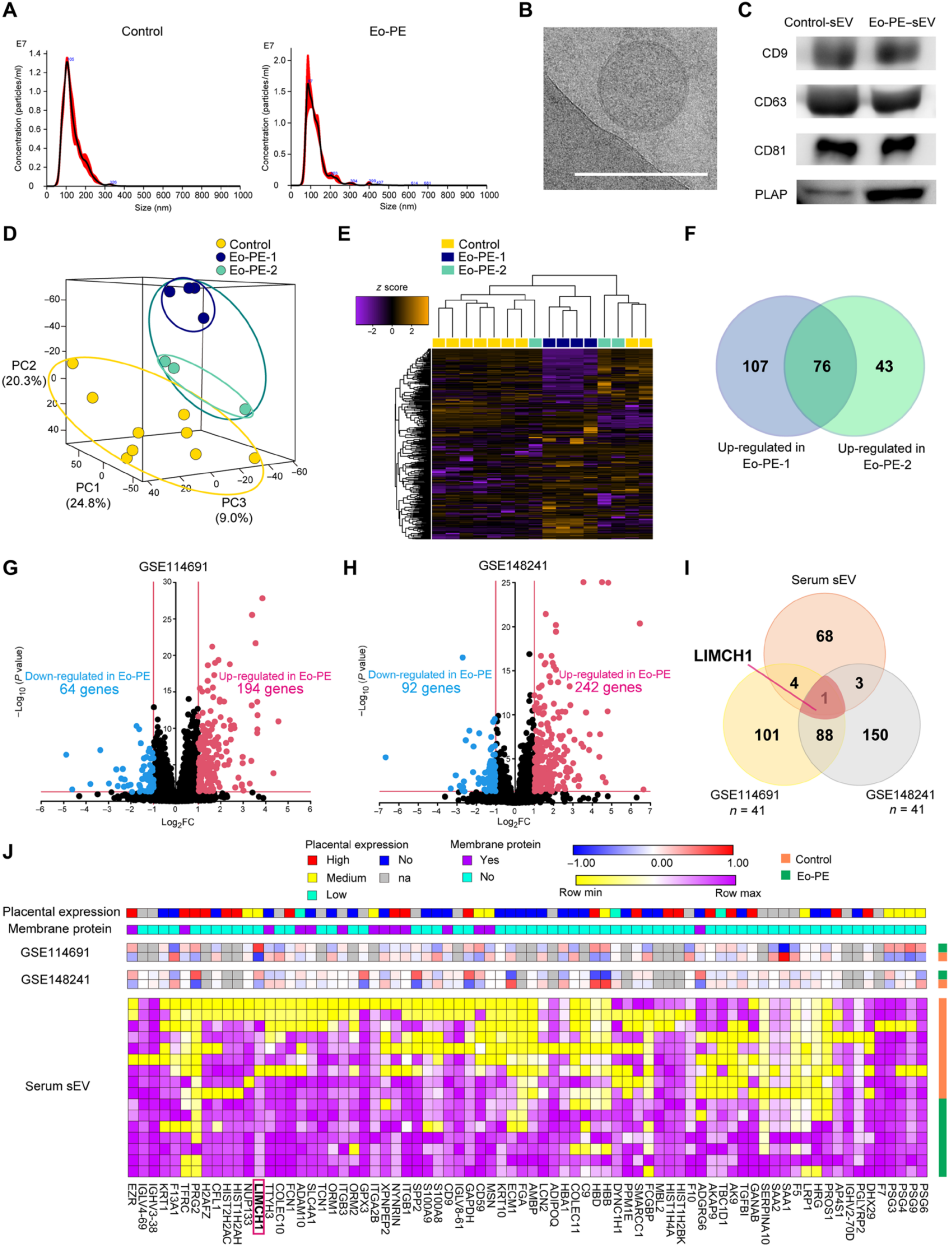
Identification of LIMCH1 as Eo-PE–associated EV protein. (**A**) Size distribution of serum sEVs from control women and women with Eo-PE was observed using NTA. (**B**) Morphology of Eo-PE serum sEV detected using TEM. Scale bar, 200 nm. (**C**) Tetraspanin (CD9, CD63, and CD81) and PLAP expression in serum sEVs. (**D**) PCA mapping for proteomic profiles of control and Eo-PE serum sEVs. In the PCA, proteomic profiles of Eo-PE EVs were divided into two clusters (Eo-PE-1 and Eo-PE-2). (**E**) Heatmap for proteomic profiles of control and Eo-PE serum sEVs. (**F**) Venn diagram showing 76 common DEGs. One hundred eighty-three and 119 proteins were up-regulated in the Eo-PE-1 and Eo-PE-2 groups compared to those in the control group, respectively. (**G**) Volcano plot showing DEGs between Eo-PE and control placentas in GSE114691 dataset. DEGs were determined as genes with an adjusted *P* value < 0.05 and absolute log_2_ fold change (log_2_FC) > 1. (**H**) Volcano plot showing DEGs between Eo-PE and control placentas in GSE148241 dataset. DEGs were determined as genes with an adjusted *P* value < 0.05 and absolute log_2_FC > 1. (**I**) Venn diagram showing common DEGs. Of the 76 up-regulated genes in Eo-PE serum sEVs, only LIMCH1 was up-regulated in the Eo-PE placenta in both datasets. (**J**) Proteomic analysis results, GEO dataset analysis results, protein location, and protein expression levels in placenta of the 76 highly expressed genes in Eo-PE serum sEVs. Data of the protein location (membrane protein or not) and the protein expression levels in placenta using UniProt database and Human Protein Atlas, respectively.

We performed serum sEV proteomic analysis to identify EV proteins associated with Eo-PE. Nine pregnant women in the control group and seven women with Eo-PE were included in the analysis. Maternal and neonatal characteristics are shown in Table 1. Nanoparticle tracking analysis (NTA), transmission electron microscopy (TEM), and Western blotting (WB) showed that the sEVs were successfully isolated (Fig. 1, A to C). Both control and Eo-PE–sEVs were positive for placental alkaline phosphatase (PLAP), indicating that serum sEVs contain placenta-derived EVs (Fig. 1C). Principal components analysis (PCA) and heatmap analysis showed that the proteomic profile of serum sEVs differed between the control and Eo-PE groups (Fig. 1, D and E). In PCA, women with Eo-PE tended to be divided into two clusters, defined as Eo-PE-1 and Eo-PE-2 (Fig. 1D). Therefore, we compared Eo-PE-1 and Eo-PE-2 with controls. Clinical information, including blood pressure in women with Eo-PE, is shown in Table 2. In the Eo-PE-2 group, complications were observed: pleural and peritoneal effusion in three women, one of whom also developed pulmonary edema. Seventy-six proteins were up-regulated in both Eo-PE groups compared to those in the control group (Fig. 1F). To narrow down the 76 proteins up-regulated in Eo-PE serum sEVs, we used two Gene Expression Omnibus (GEO) datasets and selected genes that were highly up-regulated in PE placentas. The differentially expressed genes (DEGs) of control and Eo-PE placentas in GSE114691 and GSE148241 are shown in Fig. 1 (G and H), respectively. According to the Venn diagram, only one commonly up-regulated gene, LIMCH1, was identified as an Eo-PE–associated EV protein (Fig. 1I). We also searched for protein location (membrane protein or not) and protein expression levels in the placenta using the UniProt database and Human Protein Atlas, respectively. Our proteomic analysis results, GEO dataset analysis results, protein location, and protein expression levels in the placenta of the 76 genes are shown in Fig. 1J. LIMCH1 is moderately expressed in the placenta and highly expressed in PE placentas compared with control placentas, based on two independent GEO datasets. LIMCH1 is not a membrane-associated protein. We also checked for the presence of the EV-associated proteins in categories 1 to 5 of the MISEV 2023 indications (fig. S1) (*20*).

**Table 1.** Study population characteristics.

	Control (*n* = 9)	Eo-PE (*n* = 7)	*P* value
Maternal age (years)	39.0 ± 2.6	38.6 ± 4.0	0.80
BMI (kg/m^2^)	20.9 ± 2.5	20.9 ± 0.5	0.94
Primipara	3 (33.3%)	5 (71.4%)	0.16
Gestational age at delivery (weeks)	38.8 ± 1.2	28.0 ± 3.0	<0.01
Birth weight (g)	3,036 ± 388	811 ± 329	<0.01
Male	8 (88.9%)	6 (85.7%)	0.70
Gestational age at collection (weeks)	27.4 ± 1.8	27.8 ± 2.9	0.72

Data are shown as means ± SD or *n* (%). Statistical analyses were performed using the chi-squared test or Fisher’s exact test for categorical variables and Student’s *t* test or Mann-Whitney *U* test for continuous variables according to normal or nonnormal distributions.

Eo-PE, early-onset preeclampsia; BMI, body mass index.

**Table 2.** Study population characteristics in Eo-PE pregnancies.

	Eo-PE-1 (*n* = 4)	Eo-PE-2 (*n* = 3)	*P* value
SBP (mmHg)	188 ± 30	171 ± 9	0.41
DBP (mmHg)	108 ± 19	110 ± 5	0.87
Maximum proteinuria (g/day)	7.4 ± 5.8	7.3 ± 4.0	0.98
Complication	0 (0%)	3 (100%)	0.03

Data are shown as means ± SD or *n* (%). Statistical analyses were performed using the Fisher’s exact test for categorical variables and Student’s *t* test or Mann-Whitney *U* test for continuous variables according to normal or nonnormal distributions.

Eo-PE, early-onset preeclampsia; SBP, systolic blood pressure; DBP, diastolic blood pressure.

### LIMCH1 is detected in serum sEVs, and the expression is up-regulated in Eo-PE placenta

**Fig. 2.**
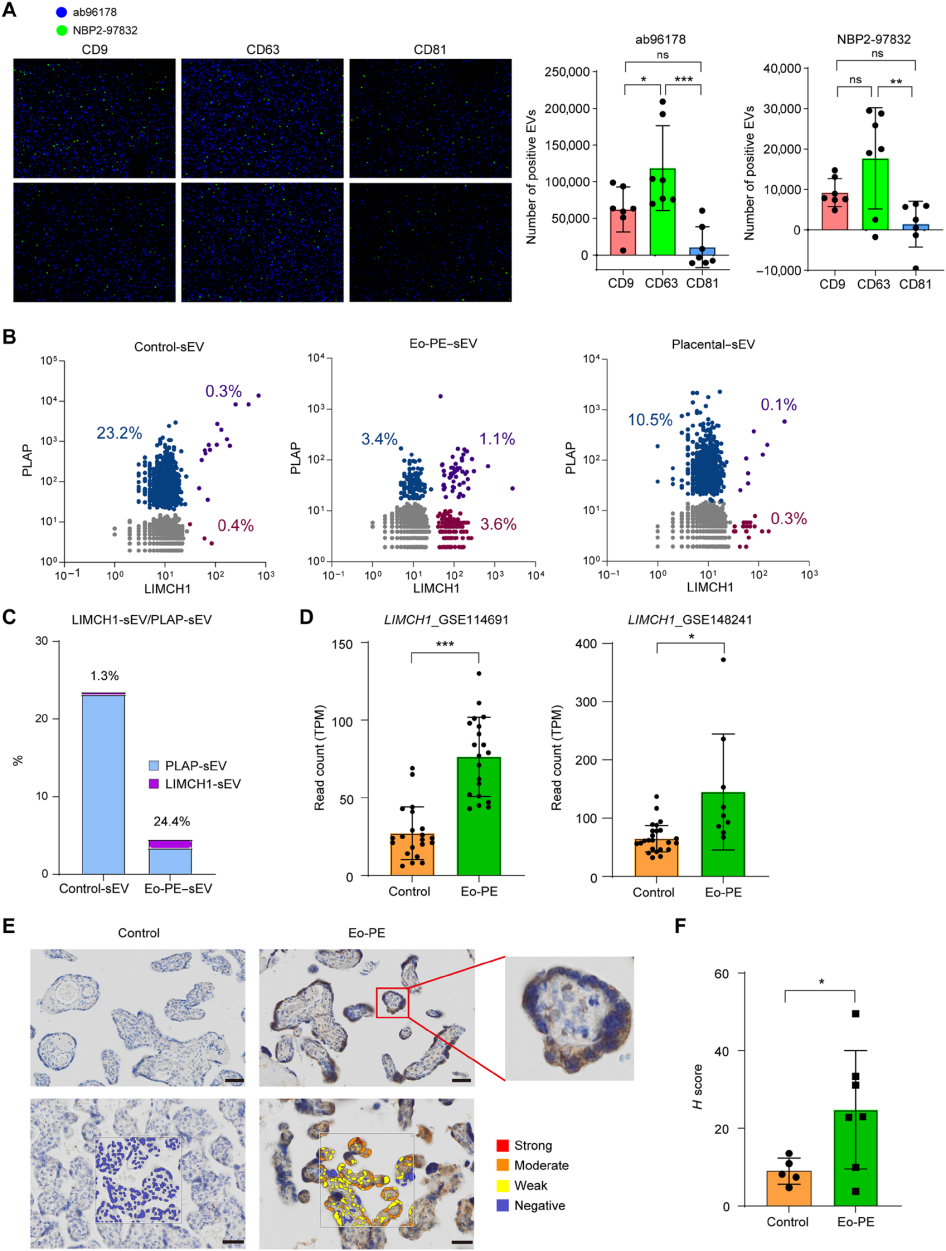
LIMCH1 is detected in serum sEVs and the expression up-regulated in Eo-PE placenta. (**A**) ExoView analysis of two women with PE using two anti-LIMCH1 antibodies (ab96178 and NBP2-97832). Right graph shows a quantitative result of ExoView analysis. Data are shown as means ± SD. ns, no significance; ***P < 0.001; **P < 0.01; *P < 0.05; Student’s *t* test. (**B**) Representative dot plots showing antibody labeling for LIMCH1 and PLAP on serum sEVs from control and PE pregnant women, as well as placental sEVs. (**C**) Light blue bars indicate the percentage of PLAP-positive EVs in serum EVs, and purple bars indicate the percentage of LIMCH1-positive EVs. The percentages shown above the bars represent the proportion of LIMCH1-positive EVs among PLAP-positive EVs. (**D**) LIMCH1 expression levels of control and Eo-PE placentas in the GEO datasets. TPM, transcripts per million. ***P < 0.001, *P < 0.05, Student’s *t* test. (**E**) Representative images of immunohistochemical staining for LIMCH1 in control and Eo-PE placentas. Scale bar, 50 μm. The bottom row shows the output of expression intensity by HALO Image Analysis software. The Eo-PE image shown in the figure corresponds to one of the panels presented in fig. S2A. (**F**) *H* score for LIMCH1 staining of the placentas. Data are shown as means ± SD. *P < 0.05, Student’s *t* test.

Next, we examined the presence of LIMCH1 in serum sEVs derived from women with PE and the expression of LIMCH1 in PE placentas. The expression of LIMCH1 in serum sEVs extracted from women with PE was confirmed using ExoView with two different antibodies (ab96178 and NBP2-97832) (Fig. 2A). LIMCH1-positive EVs were detected in serum with both antibodies. LIMCH1 was predominantly associated with CD63-positive EVs, whereas its expression was lower in CD81-positive EVs. Nanoflow cytometry (Nano-FCM) analysis revealed the presence of LIMCH1-EVs, as well as EVs coexpressing PLAP and LIMCH1 in serum, and demonstrated that the proportion of PLAP-positive EVs also positive for LIMCH1 was increased in PE (Fig. 2, B and C). Furthermore, LIMCH1 was not detected in serum EV proteomics from nonpregnant women (table S3). The mRNA level of LIMCH1 was significantly higher in Eo-PE placentas than in control placentas in both the GSE114691 and GSE148241 datasets (Fig. 2D). Since LIMCH1 was found to be up-regulated in Eo-PE placentas in the GEO datasets, we examined whether it was also up-regulated at the protein level in Eo-PE placentas in our population using immunohistochemical staining. LIMCH1 was mainly expressed in SCTs and was significantly up-regulated in PE placentas, with expression levels approximately 2.8-fold higher than those in control placentas (Fig. 2, E and F). Figure S2 shows immunostaining images of LIMCH1 in all placental samples, together with the SCT marker hCG, confirming that LIMCH1 is expressed in SCTs. To explore the mechanism underlying the increased LIMCH1 expression in PE, we examined the effect of hypoxia and found that LIMCH1 expression was up-regulated under hypoxic conditions (fig. S3).

### LIMCH1 is predominantly expressed in SCT and associated with the cell-surface interactions of the vascular wall

**Fig. 3.**
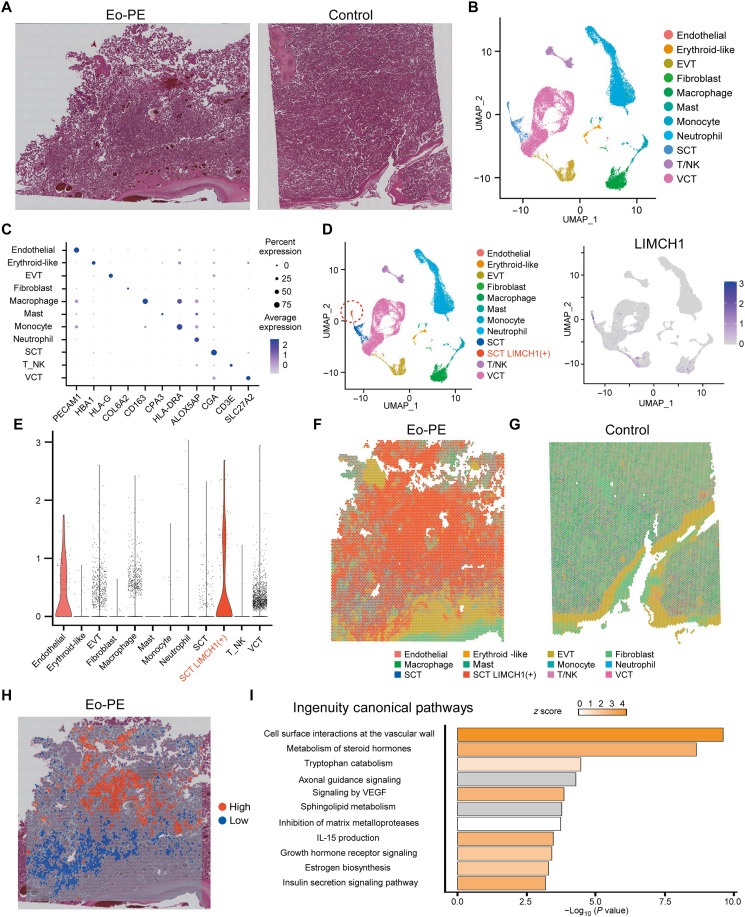
LIMCH1 is predominantly expressed in SCT and associated with the cell-surface interactions of the vascular wall. (**A**) H&E staining of control and PE placenta used in Visium transcriptomic analysis. (**B**) Uniform Manifold Approximation and Projection (UMAP) plot of the scRNA-seq dataset GSE173193 after annotation with selected marker genes. (**C**) Dot plot showing the expression patterns of selected marker genes across each cluster. (**D**) Left figure is the UMAP plot defining regions with high LIMCH1 expression within the SCT clusters as SCT LIMCH1(+). Right figure is the Feature plot of LIMCH1 expression. (**E**) Violin plot of LIMCH1 expression. (**F** and **G**) Deconvolution analysis of Visium spatial transcriptomics data. (**H**) Distribution of LIMCH1-high and LIMCH1-low spots within the SCT of the Eo-PE placenta. (**I**) Significantly dysregulated pathways identified by Ingenuity Pathway Analysis. For both the Eo-PE and control placentas, the histological H&E images (A) and the Visium spatial transcriptomic analyses [(F) for Eo-PE, (G) for control, and (H) for Eo-PE)] and derived from the same respective specimens.

**Fig. 4.**
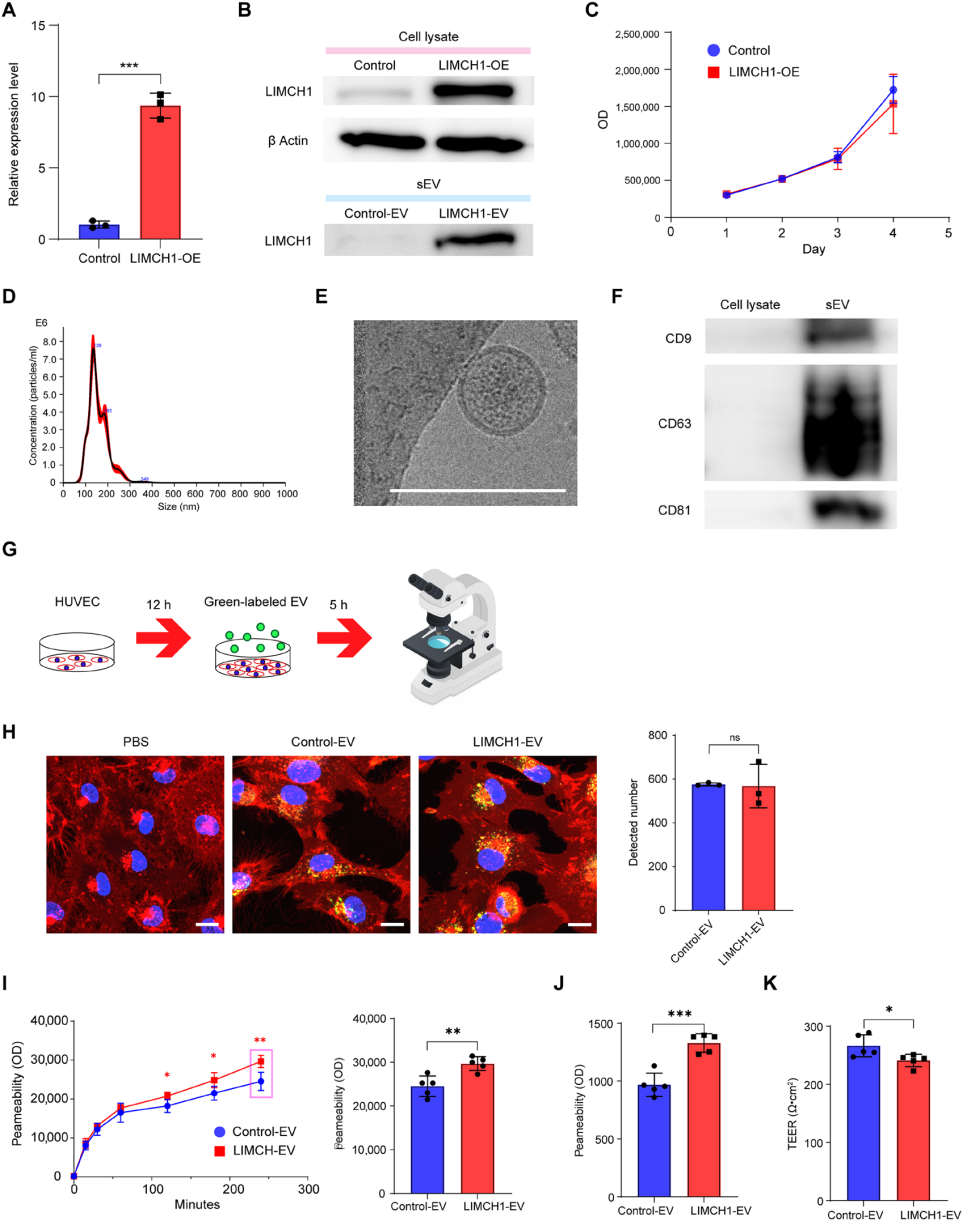
LIMCH1-EVs increase transendothelial permeability. (**A**) Generation of LIMCH1-OE JAR cells. LIMCH1 expression was measured by RT-qPCR with glyceraldehyde 3-phosphate dehydrogenase (*GAPDH*) as an internal control. ***P < 0.001, Student’s *t* test. (**B**) Immunoblot analysis of LIMCH1 and β-actin in control, LIMCH1-OE JAR cells, and their derived EVs. (**C**) Cell viability of JAR-control and JAR-LIMCH1-OE cells. Data are shown as means ± SD. (**D**) Size distribution of JAR-derived sEVs using NTA. (**E**) Morphology of JAR-sEV detected using TEM. Scale bar, 200 nm. (**F**) Immunoblot analysis of tetraspanin markers (CD9, CD63, and CD81) in JAR cell lysates and sEVs. (**G**) Schematic representation of the experimental protocol for EV uptake into HUVECs. CellMask Green–labeled EVs were added to HUVECs and observed after 5 h (hours). (**H**) Representative confocal microscopy images. EVs were labeled with CellMask Green, and cell membrane and nuclei were stained with CellMask Deep Red (red) and Hoechst (blue), respectively. Scale bar, 10 μm. Right graph shows quantitative analysis using NIS-Elements AR. Data are shown as means ± SD. ns, no significance; Student’s *t* test. Biological *n* = 3 per condition. Images represent cropped regions of fig. S5. (**I** and **J**) In vitro permeability assay using 40-kDa (I) and 70-kDa (J) FITC-dextran. HUVECs cultured on transwell inserts were treated with 6 μg of Control-EVs or LIMCH1-EVs. FITC-dextran was added after 24 hours, fluorescence in the lower chamber was measured as the optical density (OD), and OD after 4 hours was compared. Data are shown as means ± SD. *P < 0.05, **P < 0.01, ***P < 0.001, Student’s *t* test. (**K**) TEER measurements. Data are shown as means ± SD. *P < 0.05, Student’s *t* test. Data are representative of at least three independent experiments [(I) to (K)].

Next, to investigate the key pathways involving LIMCH1 in the development of PE, we performed a spatial transcriptome analysis. Figure 3A shows the hematoxylin and eosin (H&E) staining of the control and PE placentas used for spatial transcriptomic analysis. The PE placenta showed some coagulation necrosis of the villi, which is typically observed in PE. We integrated two PE cases and two control cases from a single-cell RNA sequencing (scRNA-seq) dataset of the placenta (GSE173193) and performed unsupervised clustering, identifying 27 clusters (fig. S4A). Using known marker genes, we identified 11 cell types, which were consistent with established marker gene expression patterns (Fig. 3, B and C). Consistent with the immunohistochemistry results, LIMCH1 was predominantly expressed in SCT, and within the SCT, regions with high and low LIMCH1 expression were observed. Therefore, the SCT was further subdivided, with the high-expression subset defined as SCT LIMCH1(+) (Fig. 3, D and E). We further confirmed that LIMCH1 expression was higher in Eo-PE placentas than in control placentas (fig. S4, B and C). The results of the deconvolution analysis are shown in Fig. 3 (F and G). Each spots contain approximately 10 cells. In the Eo-PE placenta, SCT LIMCH1(+) cells were the most abundant. To explore the pathways associated with LIMCH1, we compared SCT LIMCH1-high and SCT LIMCH1-low spots (Fig. 4H). Spots in which the proportion of SCT LIMCH1(+) cells ranked in the top 10% were defined as SCT LIMCH1-high, whereas spots in which the proportion of SCT cells (i.e., LIMCH1-low SCTs) ranked in the top 10% were defined as SCT LIMCH1-low. The cell-type proportions of SCT and SCT LIMCH1(+) in the Eo-PE placenta are shown in fig. S4D. The top significantly dysregulated pathways between SCT LIMCH1-low and SCT LIMCH1-high were “cell-surface interactions at the vascular wall,” followed by “metabolism of steroid hormones” and “tryptophan catabolism” (Fig. 3I). In summary, LIMCH1 is mainly expressed in synscytiotrophoblasts and is associated with cell-surface interactions at the vascular wall.

### LIMCH1-EVs increase transendothelial permeability

We then examined the effects of LIMCH1-enriched sEVs on endothelial cells using human umbilical vein endothelial cells (HUVECs) because LIMCH1 expression is associated with the cell-surface interaction of the vascular wall. To examine the effects of LIMCH1-enriched sEVs on HUVECs, we generated LIMCH1-overexpressing (LIMCH1-OE) JAR cells. We confirmed the successful establishment of LIMCH1-OE JAR cells by reverse transcription quantitative polymerase chain reaction (RT-qPCR) and WB (Fig. 4, A and B). The proliferation of LIMCH1-OE JAR cells was comparable to that of the control JAR cells, as assessed by the CellTiter-Glo Luminescent Cell Viaility Assay (Fig. 4C). In addition, NTA, TEM, and WB confirmed the successful isolation of JAR-sEVs (Fig. 4, D to F). We confirmed that LIMCH1 was enriched in LIMCH1-EVs (Fig. 4B). Next, the uptake of control- and LIMCH1-EVs into HUVECs was observed using a confocal microscope (Fig. 4H and fig. S5). There was no difference in the number of detected particles between the control- and LIMCH1-EVs. Administration of LIMCH1-EVs did not affect the proliferation or tube-formation ability of HUVECs (fig. S6, A and B). However, administration of LIMCH1-EVs significantly increased endothelial permeability compared with Control-EVs, as demonstrated by leakage of 40-kDa fluorescein isothiocyanate (FITC)–dextran (Fig. 4I) and 70-kDa FITC-dextran (Fig. 4J). Similar results were obtained for an overexpressing strain generated from another clone (fig. S7). Furthermore, transendothelial electrical resistance (TEER) decreased following LIMCH1-EV administration, consistent with the findings of the FITC-dextran permeability assays (Fig. 4K). To confirm the consistency of this finding, we generated LIMCH1-OE BeWo cells by the same method as in the JAR except for the concentration of puromycin (2.5 μg/ml). NTA, TEM, and WB revealed the successful isolation of BeWo-sEVs (fig. S8, A to C). There was no difference in the endothelial permeability between BeWo–Control-EVs and BeWo–LIMCH1-EVs (fig. S8D). To explore the reason for this inconsistency of the results, we found that sEVs derived from BeWo cells showed less uptake into HUVECs compared with those of JAR cells (fig. S8, E and F) although the number of particles per 1 μg of EV did not differ between BeWo- and JAR-sEVs (fig. S8G). Furthermore, proteomic analyses of BeWo- and JAR-sEVs revealed markedly distinct profiles, which may have contributed to the observed differences in EV uptake (fig. S8, H to K).

### LIMCH1-EVs disrupt vascular endothelial cell-cell junction by decreasing ZO-1 expression

**Fig. 5.**
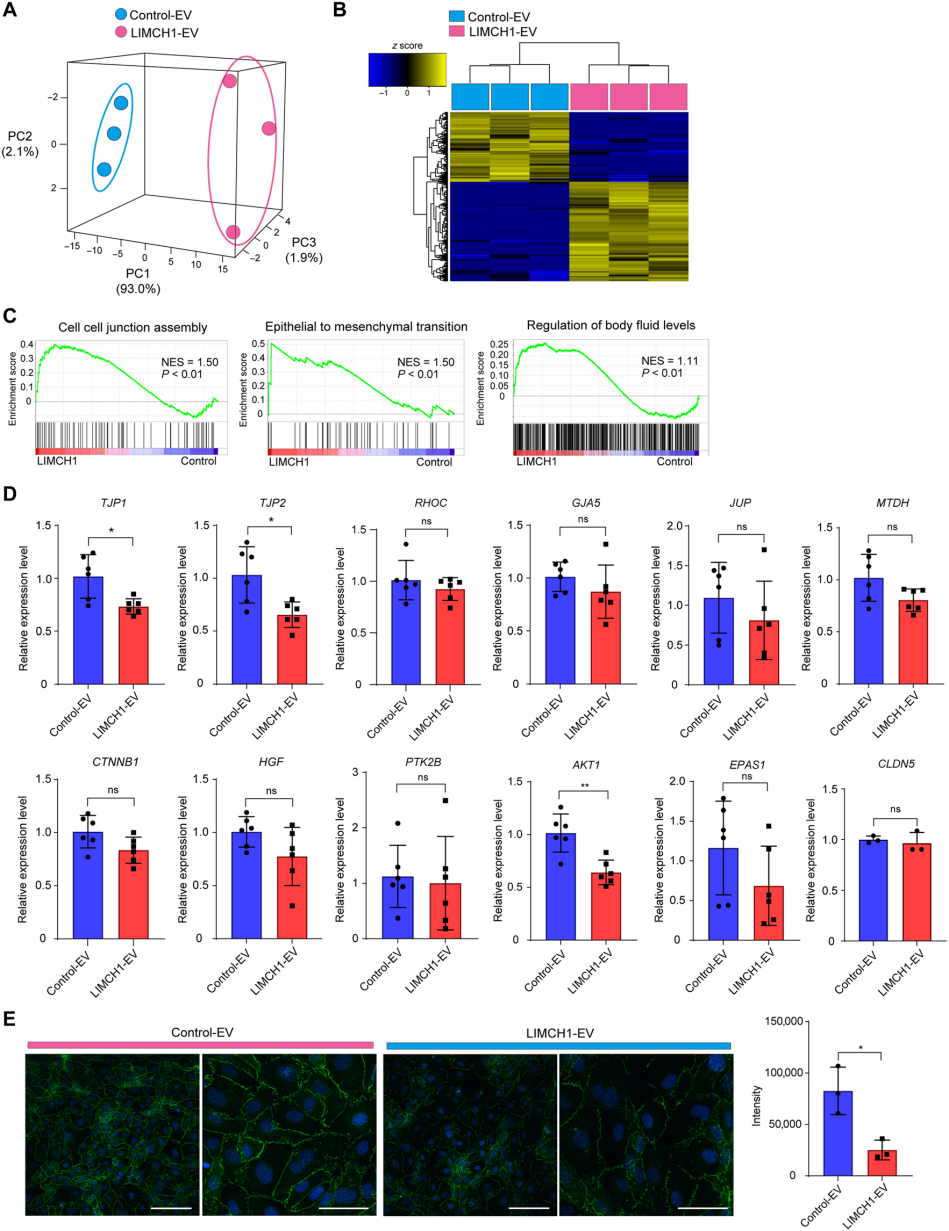
LIMCH1-EVs disrupt vascular endothelial cell-cell junction by decreasing ZO-1 expression. (**A**) PCA mapping for mRNA profiles of Control-EV– and LIMCH1-EV–treated HUVECs. (**B**) Heatmap showing mRNA profiles of Control-EV– and LIMCH1-EV–treated HUVECs. (**C**) GSEA of Control-EV–treated HUVECs versus LIMCH1-EV–treated HUVECs, highlighting destructive phenotypes. NES, normalized enrichment score. The *P* values in the graphs were calculated by GSEA. (**D**) Validation of genes included in multiple enrichment gene set by real-time quantitative PCR. The expression was measured and *GAPDH* used as an internal control. *P < 0.05, **P < 0.01, and ns, no significance; Student’s *t* test. (**E**) Representative images using confocal microscopy. After treatment with Control-EVs or LIMCH1-EVs for 24 hours, HUVECs were analyzed by immunofluorescence for ZO-1 (green). The nuclei were stained with 4′,6-diamidino-2-phenylindole (DAPI) (blue). Right graph shows a quantitative result of signal intensity using NIS-Elements AR Analysis program. Data are shown as means ± SD. *P < 0.05, Student’s *t* test. Data reflect biological *n* = 3 per condition.

RNA-seq was performed on HUVEC to investigate the molecular mechanisms associated with increased endothelial permeability induced by LIMCH-EVs. Figure 5 (A and B) shows that LIMCH1-EVs altered the gene expression profile of HUVECs. Gene set enrichment analysis (GSEA) was performed to identify the effects of LIMCH1-EVs on HUVECs. Several functions and processes, such as cell-cell junctions, epithelial-to-mesenchymal transition (EMT), and regulation of body fluid levels, were significantly different between Control-EV and LIMCH1-EV-treated HUVECs (Fig. 5C). We selected genes that were included in the multiple enrichment gene set to validate gene expression differences between Control-EV and LIMCH1-EV–treated HUVECs using RT-qPCR. Among 11 selected genes, the mRNA level of tight junction protein 1 (*TJP1*), tight junction protein 2 (*TJP2*), and AKT serin/threonine kinase 1 (*AKT1*) were significantly decreased in the HUVECs treated by LIMCH1-EVs (Fig. 5D). Because tight junction regulation emerged as a major enriched pathway, we additionally assessed the expression of Claudin-5—a key tight junction component—and found that its mRNA level remained unchanged following LIMCH1-EV exposure. *TJP1* encodes ZO-1, which is known to be expressed at tight junctions of endothelial cells, and its decreased expression has been reported to be associated with brain and pulmonary edema (*21*, *22*). Furthermore, we confirmed that ZO-1 was expressed at tight junctions in HUVECs and that the protein level of ZO-1 was significantly decreased after the administration of LIMCH1-EVs by immunofluorescent staining (Fig. 5E).

### LIMCH1-EVs increase lung vascular permeability in vivo

**Fig. 6.**
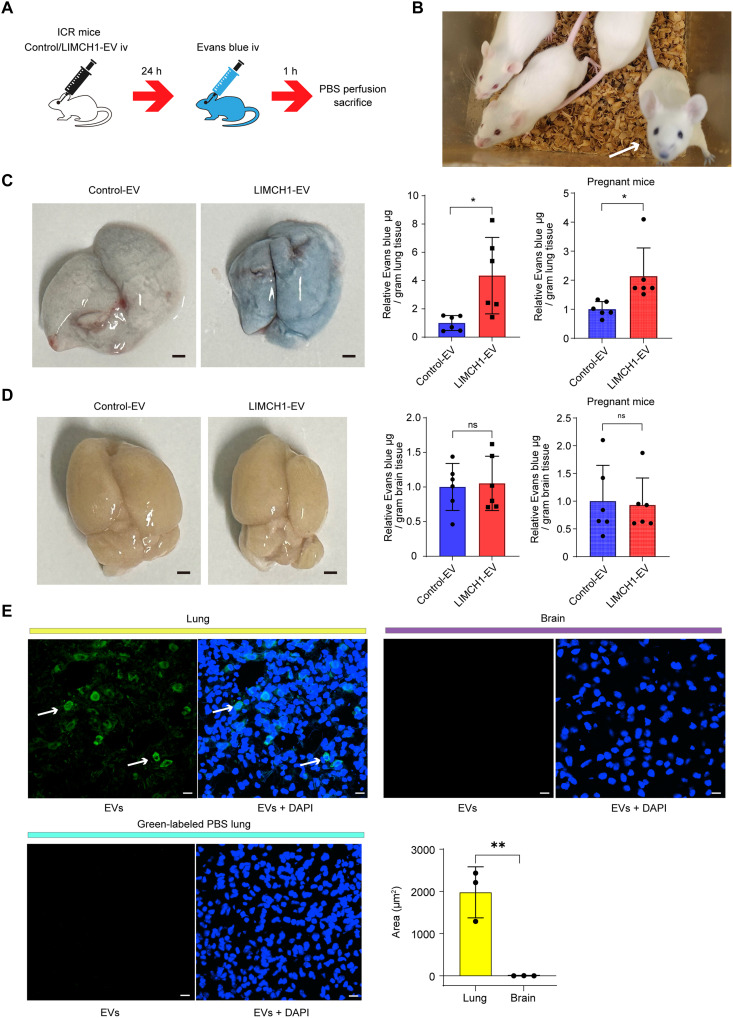
LIMCH1-EVs increase lung vascular permeability in vivo*.* (**A**) Schematic representative protocol of the Evans blue permeability assay in vivo. ICR mice were administered 100 μl PBS containing 10 μg of Control-EVs (*n* = 6) or LIMCH1-EVs (*n* = 6) retro-orbitally. Twenty-four hours later, 100 μl of 2% Evans blue dye was injected retro-orbitally. Lung and brain tissues were collected 1 hour after injection of Evans blue dye. (**B**) Images of the mice. White arrow shows a mouse several minutes after Evans blue injection. (**C**) Representative image of the lungs collected from Control-EV–injected and LIMCH1-EV–injected mice. Scale bar, 1 mm. Graphs showing the quantitative results of Evans blue permeability assay (left, nonpregnant mice; right: pregnant mice). Data are shown as means ± SD. *P < 0.05, Student’s *t* test. (**D**) Representative image of the brain collected from Control-EV–injected and LIMCH1-EV–injected mice. Scale bar, 1 mm. Graphs showing the quantitative results of Evans blue permeability assay (left, nonpregnant mice; right, pregnant mice). Data are shown as means ± SD. ns, no significance; Student’s *t* test. (**E**) Representative images using confocal microscopy. EVs were labeled with CellMask Green, and ICR mice were administered CellMask Green–labeled PBS (*n* = 1) or CellMask Green–labeled 10 μg of JAR EVs (*n* = 3). Nuclei were counterstained blue with DAPI. White arrows indicate cells with abundant EV uptake. Scale bar, 10 μm. The images shown here represent magnified regions of the corresponding images presented in fig. S9A. Bottom right graph shows a quantitative result of NIS-Elements AR Analysis program. Data are shown as means ± SD. **P < 0.01, Student’s *t* test.

To determine whether LIMCH1-EVs increased endothelial permeability in vivo, we used Evans blue dye, which can estimate vascular leakage. The ears, tail, and limbs turned blue soon after intravenous administration of Evans blue (Fig. 6B). Greater Evans blue leakage into the lungs was observed in both pregnant and nonpregnant mice administered LIMCH1-EVs than in those administered Control-EVs, whereas Evans blue leakage into the brain was comparable between the two groups (Fig. 6, C and D). To determine the reason for the lack of leakage into the brain, we compared the uptake of CellMask Green–labeled EVs in the lungs, brains, and kidneys. Uptake of labeled EVs into cells was clearly observed in the lungs; however, little cellular uptake was observed in the brain and kidneys (Fig. 6E and fig. S9). No cellular uptake was observed after the administration of CellMask Green–labeled phosphate-buffered saline (PBS) (Fig. 6E and fig. S9). In addition, there were no significant differences in blood pressure, urinary protein levels, or embryonic weight between the two groups (fig. S10).

## DISCUSSION

The major findings of this study are that LIMCH1 is an Eo-PE–related EV protein and that LIMCH1-EVs are associated with endothelial dysfunction, a central feature of PE. In addition, we demonstrated that LIMCH1-EVs increased vascular permeability both in vitro and in vivo, which was caused by the down-regulation of ZO-1 expression at tight junctions of the vascular endothelium. These findings provide previously unknown insights into the mechanisms underlying PE-related pulmonary and generalized edema.

Recent evidence has demonstrated that placenta-derived EVs play a crucial role in pregnancy and are involved in the development of pregnancy-related complications, including PE. The central pathophysiology of PE is systemic endothelial dysfunction, and recent studies have shown that serum sEVs derived from women with PE induce vascular endothelial damage. Leon *et al.* (*17*) reported that PE serum sEVs promoted vascular permeability in a human cerebral microvascular endothelial cell line and disrupted the BBB in vivo. Chang *et al.* (*23*) reported that pregnant mice injected with PE serum EVs develop a PE-like phenotype. Although the latter study suggested that the PE-like phenotype may be due to sFlt-1 and soluble endoglin contained in EVs, the detailed mechanisms remain to be elucidated. In this study, we identified LIMCH1-EVs as contributors to endothelial dysfunction associated with PE.

LIMCH1 is a cytoplasmic protein associated with actin stress fibers and is involved in cell adhesion and migration (*24*). High expression is associated with good prognosis in lung cancer and renal cell carcinoma, while high expression is associated with poor prognosis and metastasis in breast cancer (*25*). LIMCH1 has been reported to be up-regulated in PE placentas; however, its detailed function and regulation in the pathogenesis of PE remain to be investigated (*26*, *27*). Consistent with previous studies, LIMCH1 was up-regulated in Eo-PE placentas in both the GEO datasets and our samples (*28*, *29*). Furthermore, LIMCH1 was predominantly expressed in SCTs, which are the primary source of placenta-derived EVs (*14*). Therefore, it is highly plausible that up-regulated expression of LIMCH1 in SCTs of PE placentas results in an increased release of LIMCH1-EVs into maternal circulation. LIMCH1 expression was elevated under hypoxic conditions, suggesting that placental ischemia could trigger the release of LIMCH1-EVs from the placenta.

According to spatial transcriptomic analysis, the top significantly dysregulated pathways between LIMCH1-low and LIMCH1-high cluster were cell-surface interactions at the vascular wall, followed by metabolism of steroid hormones and tryptophan catabolism. Decreased synthesis of steroid hormones (*30*) and decreased tryptophan content in the placenta have been reported to be associated with PE (*31*). Although the involvement of LIMCH1 in these signaling pathways remains to be elucidated, it has been suggested that LIMCH1 contributes to PE through multiple pathways. Because LIMCH1 is possibly associated with cell-surface interactions at the vascular wall, we examined the effect of LIMCH1-EVs on HUVECs.

Increased vascular permeability is a characteristic vascular response in PE, resulting in systemic edema, pulmonary edema, and PRES (*32*–*34*). Cytokines, oxidants, proteases, and vascular endothelial growth factor has been reported to be involved in the mechanism of vascular permeability, but there is still no definitive answer (*35*–*38*). In this study, we focused on EVs and found that while the administration of LIMCH1-EVs did not affect the proliferation or tube formation ability of HUVECs, LIMCH1-EVs promoted the transendothelial permeability of HUVECs and increased pulmonary vascular permeability in vivo. On the basis of several previous reports, LIMCH1 has been implicated in the regulation of cell migration and may also be indirectly involved in angiogenic processes (*24*). However, direct experimental evidence linking LIMCH1 to angiogenesis remains limited, and the functional role of LIMCH1, particularly LIMCH1-EVs has not been clearly defined. Accordingly, our findings may reflect a more specific effect of LIMCH1-EVs on endothelial barrier integrity rather than on angiogenic pathways. Our findings suggest that LIMCH1-EVs may be associated with pulmonary endothelial dysfunction relevant to pulmonary edema. Pulmonary edema is an acute, life-threatening condition, which is the second most common cause of death in pregnancies complicated by hypertension (*39*, *40*). The reason for the increased vascular permeability in the lungs, but not in the brain, in our experimental model is assumed to be that fewer EVs were taken into the brain because almost all EVs were trapped in the lungs when they were administered via intravenous injection (Fig. 6E). Even in the kidney, where the vascular barrier is weak, only minimal EV uptake was observed, suggesting that most EVs are sequestered in the lungs. Consistent with our study, it has been reported that EVs are not distributed in the brain after intravenous injection but only after intracardiac injection (*41*). However, intracardiac injection is a challenging technique that cannot be performed accurately and uniformly (*42*). Disrupted BBB by intravenous EV injection in vivo was reported by Leon *et al.*; however, 20 times more EVs (200 μg EVs per mouse) were used in their study than in our study (*17*). Administration of LIMCH1-EVs did not result in any changes in blood pressure, urinary protein levels, or embryonic weight. These findings indicate that LIMCH1-EVs do not directly induce the systemic manifestations of PE but instead contribute primarily to the local pathological mechanism of increased pulmonary endothelial permeability. Therefore, although LIMCH1-EVs alone are insufficient to recapitulate the entire pathophysiology of PE, they may be involved in pathways contributing to a specific aspect of the disease. This interpretation is also compatible with the notion that hypertension in PE may be driven primarily by vasoconstrictive pathways—such as reduced nitric oxide bioavailability and increased endothelin-1—rather than by vascular hyperpermeability alone (*38*). The absence of proteinuria in our model is also consistent with this view as only minimal uptake of JAR-EVs was observed in the kidneys, suggesting that insufficient renal exposure to EV may have prevented the development of glomerular injury.

In our study, we observed no difference in endothelial permeability between BeWo–Control-EVs and BeWo–LIMCH1-EVs, in contrast to the results obtained with JAR-derived EVs. Our imaging analysis revealed that BeWo-derived EVs exhibited substantially lower internalization by HUVECs, suggesting that their attenuated permeability effect is likely attribute to reduced cellular uptake. Given that EV uptake depends on a variety of surface molecules—including tetraspanins, integrins, and adhesion-related proteins—differences in the surface protein composition of BeWo-derived EVs may impair their ability to interact with endothelial receptors and undergo efficient endocytosis (*43*). In addition, our proteomic analysis demonstrated marked differences in the overall protein cargo between JAR- and BeWo-derived EVs, which may contribute to their divergent functional effects. However, we acknowledge that our current data do not directly identify specific surface molecules responsible for the reduced internalization of BeWo-EVs. Future studies will be required to elucidate the precise mechanisms governing EV uptake and functional specificity.

We found that the suppressed expression of the tight junction protein ZO-1 in endothelial cells induced by LIMCH1-EVs is presumed to be a key driver of increased vascular permeability. ZO-1 is ubiquitously expressed in the tight junctions of epithelial and endothelial cells, and its decreased expression has been associated with brain and pulmonary edema due to tight junction dysfunction (*21*, *22*, *44*). Regarding the relationship between ZO-1 and EVs, Zhou *et al.* (*45*) reported that the EV-mediated transfer of miR-105 suppressed ZO-1 expression in endothelial cells and resulted in the loss of cell-cell adhesion. LIMCH1-EV administration also significantly reduced the expression of Akt1. The Akt pathway and the phosphatidylinositol 3-kinase R3/nuclear factor κB pathway have been reported to regulate the expression of ZO-1, and it is possible that these pathways were activated following the administration of LIMCH1-EVs (*46*, *47*). In addition, the GSEA results showed that several functions or processes, such as EMT and regulation of body fluid levels, were significantly different between Control-EV– and LIMCH1-EV–treated HUVECs. EMT of endothelial cells has been reported to cause cardiovascular diseases (*48*). Higher body fluid levels are typical features of PE (*49*). These findings indicate that LIMCH1-EVs not only induce increased vascular permeability but also may contribute to features such as increased body fluid levels or cardiovascular alterations observed in PE. In addition, although Claudin-5 is a key tight junction component regulating endothelial barrier function, its mRNA expression was not altered by LIMCH1-EV treatment. This suggests that LIMCH1-EVs may disrupt tight junction organization primarily through down-regulation of ZO-1 rather than through transcriptional suppression of Claudin-5.

This study has two strengths. First, we focused on the EV-mediated mechanism of PE, which has not been well studied, and identified LIMCH1-EVs as mediators. Next, this study provided deep insights into the pathophysiology of PE because we focused on vascular permeability, which is a characteristic vascular response in PE. In particular, it is worthwhile to verify that PE-related EVs increase pulmonary vascular permeability in vivo. Our study has several limitations. First, we were unable to directly demonstrate that LIMCH1-EVs in the maternal circulation were of placental origin. However, LIMCH1 was moderately expressed in the placenta according to the Protein Atlas database and was highly up-regulated in PE placentas. Moreover, immunohistochemical staining and spatial transcriptomic analysis demonstrated that LIMCH1 is predominantly expressed in SCTs, and Nano-FCM analysis revealed EVs coexpressing PLAP and LIMCH1, with an increased number of PLAP- and LIMCH1-positive EVs in PE. Together, these findings strongly suggest that LIMCH1-EVs are of placental origin. Second, although we examined pulmonary vascular permeability in vivo, we could not assess the effects of LIMCH1-EVs on the development of cerebral edema or PRES because little EV uptake into the brain was observed using our method, and we were also unable to directly evaluate the effects of serum EVs on tight junctions. However, a previous study reported that serum EVs from women with PE induced vascular endothelial dysfunction compared with those from normotensive pregnant women (*16*). Third, the clinical correlation analyses were based on a small cohort of women with Eo-PE, and only one woman presented with pulmonary edema (with two additional women exhibiting pleural effusion). Therefore, these data are insufficient to establish a robust association between circulating LIMCH1-EV levels and pulmonary compromise, and the clinical findings should be interpreted with caution. Fourth, our RNA-seq analysis was conducted using a limited number of samples, which inevitably reduces the statistical power and may limit the reproducibility of the findings. To mitigate this limitation, we performed validation experiments using additional samples to confirm the key transcriptomic changes. Nevertheless, these results should be interpreted with caution, and validation in larger, independent cohorts is warranted. Fifth, our in vitro experiments were performed using HUVECs as a general endothelial model. Although HUVECs are widely used and well established for evaluating endothelial barrier function—particularly in PE-related research—they do not fully recapitulate the organ-specific characteristics of pulmonary microvascular endothelial cells (PMVECs). Given that the in vivo phenotype was predominantly observed in the lungs, the use of PMVECs would provide greater physiological relevance. However, we were unable to access PMVECs. Future studies incorporating PMVEC-based assays will be necessary to delineate tissue-specific endothelial responses and to validate the mechanistic differences suggested by our HUVEC data.

In summary, we demonstrated that LIMCH1-EVs in women with PE increase endothelial permeability, a feature associated with PE. We believe that these findings will lead to improvements in the clinical outcomes of women with PE. Further studies are necessary to deepen our understanding of LIMCH1-EVs as potential markers of severe PE and as therapeutic targets for Eo-PE.

## MATERIALS AND METHODS

### Study population and serum sample collection

Serum samples were collected from patients with Eo-PE after diagnosis (*n* = 7) and from control pregnant women (*n* = 9) who provided written informed consent and delivered at Nagoya University Hospital between January 2014 and March 2022. PE was diagnosed when women developed hypertension (≥140/90 mmHg) during pregnancy complicated by maternal organ damage or uteroplacental insufficiency according to the guideline (*50*). The PE and control groups were matched for maternal age, body mass index, and gestational age at the time of blood collection. After peripheral venous blood sample collection, blood samples were immediately centrifuged at 3000 rpm for 10 min at 4°C and the serum was kept at −80°C until use. The study protocol was approved by the Institutional Ethics Board of Nagoya University (approval number: 2017-0302, approval date: 09 November 2017).

### Cell culture

Human choriocarcinoma cell lines (JAR and BeWo) were purchased from the American Type Culture Collection (ATCC, Manassas, VA, USA). JAR was cultured in Dulbecco’s modified Eagle’s medium (DMEM, 4500 mg/liter glucose, Nacalai Tesque Inc., Kyoto, Japan) supplemented with 10% fetal bovine serum (FBS) and 1% penicillin-streptomycin at 37°C in 5% CO_2_. BeWo was cultured in Ham’s F-12 medium (FUJIFILM Wako Pure Chemical Corporation, Osaka, Japan) supplemented with 10% FBS and 1% penicillin-streptomycin at 37°C in 5% CO_2_. HUVECs were obtained from ATCC and cultured in endothelial cell growth medium (EGM-2 supplemented with a bullet kit; catalog no. CC-3162; Lonza, USA) at 37°C in 5% CO_2_.

### EV purification and analysis

The EV isolation method used in this study adhered to the standard principles of the International Society for Extracellular Vesicles (*20*, *51*, *52*). We isolated sEVs in our experiments. The cells were washed with PBS, and the culture medium was replaced with advanced DMEM (Thermo Fisher Scientific Inc., USA) for JAR cells and Ham’s F-12 (FUJIFILM Wako Pure Chemical Corporation) supplemented with 2% exosome-depleted FBS (EXO-FBS-50A-1, SBI, Japan) for BeWo cells. After incubation for 48 hours, the conditioned medium (CM) was collected and centrifuged at 2000*g* for 10 min at 4°C. To thoroughly remove cellular debris, the supernatant was filtered through a 0.22-μm filter (Corning, USA). CM was used for EV isolation.

To prepare serum sEVs, 1 ml of each serum sample from patients was filtered using a 0.22-μm filter (Millex-GV 33 mm, Millipore, USA) and ultracentrifuged at 45,000 rpm using an MLS-50 rotor (Beckman Coulter Inc., USA) for 70 min at 4°C; CM was ultracentrifuged at 32,000 rpm using an SW 32 Ti rotor (Beckman Coulter) for 120 min at 4°C. The pellet was washed again with PBS, ultracentrifuged under the same conditions, and resuspended in PBS. The protein concentration of the putative EV fraction was determined using a Quant-iT Protein Assay with a Qubit 2.0 Fluorometer (Invitrogen, USA). In the in vitro experiments, EV doses were normalized by total protein content and administered at a concentration of 30 μg/ml. This dose was selected on the basis of concentrations commonly used in previous studies evaluating EV-mediated endothelial barrier regulation (typically 10 to 30 μg/ml) and therefore represents a physiologically and experimentally validated range (*53*, *54*). To determine the size distribution of EVs, NTA was carried out using a Nanosight system (Quantum Design, Tokyo, Japan) on samples diluted 500- to 1000-fold with PBS.

### Electron microscopy

The isolated sEVs were visualized using phase-contrast TEM (Terabase Inc., Okazaki, Japan). TEM was performed at our institute for BeWo-EVs using the following method: In brief, 10 μl of EVs solubilized in ultrapure water were dropped on to Parafilm (Bemis Company Inc., Neenah, WI, USA). A carbon-coated Formvar copper grid (catalog no. 645, Nisshin EM Co., Japan) was placed on the droplet to immerse its coated side and incubated for 30 s at room temperature. After negative staining with a 2% uranium diacetate solution for 1 min, the excess solution was dabbed with a piece of filter paper, and the samples were dried at room temperature. The grid was visualized at increasing magnifications up to 50 K using the JEM-2100 (JEOL Co., Japan).

### LC-MS/MS analysis

Approximately 1 ml of each serum sample was filtered using a 0.22-μm filter (Millex-GV 33 mm) and ultracentrifuged at 45,000 rpm using an MLS-50 rotor (Beckman Coulter) for 70 min at 4°C and resuspended in 60 μl of PBS. Albumin and immunoglobulin G (IgG) levels were reduced using the ProteoExtract Albumin/IgG Removal Kit (Merck & Co. Inc., USA) and then ultracentrifuged again at 45,000 rpm using an MLS-50 rotor (Beckman Coulter) for 70 min at 4°C. The pellet was resuspended in 100 μl of lysis solution contained in EasyPep Mini MS Sample Prep Kit (catalog no. A40006, Thermo Fisher Scientific), trypsin-digested after reduction and alkylation, according to the manufacturer’s protocol. Approximately 10 μg of EVs from JAR-sEVs and BeWo-sEVs were processed in the same manner using the EasyPep Mini MS Sample Prep Kit. The samples were analyzed using an Advanced Liquid Chromatography system (Bruker Corporation, Billerica, MA) equipped with a MonoCap C18 0.1 mm in diameter and 150 mm in length (GL Sciences Inc., Japan). Reversed-phase chromatography was performed with a linear gradient (0 min, 5% B; 100 min, 40% B) of solvent A (2% acetonitrile with 0.1% formic acid) and solvent B (95% acetonitrile with 0.1% formic acid) at an estimated flow rate of 300 nl/min. A precursor ion scan was performed with a mass-to-charge ratio (*m*/*z*) of 400 to 1600 before MS/MS analysis. The raw data were processed using Proteome Discover 1.4 (Thermo Fisher Scientific) in conjunction with the MASCOT search engine, version 2.6.0 (Matrix Science Inc., Boston, MA, USA) for protein identification. A total of 966 human proteins were identified, and 356 proteins detected in half of the cases were extracted for analysis. Using RStudio (RStudio Inc., Boston, MA, USA) and R software (ver. 4.0.3), the data were converted to base 2 logarithms and normalized using the quantile method. To calculate and visualize PCA, we used the prcomp and plot3d functions of the rgl package (ver. 1.2.8). The heatmap.2 function in the gplots package (ver. 3.1.3) was used to convert the data into *z* scores. To identify the up-regulated proteins in Eo-PE, we used a mean log_2_ fold change (log_2_FC) of >1 as the cutoff.

### Identification of Eo-PE–related EV protein

To identify the proteins up-regulated in Eo-PE serum sEVs, we used two GEO datasets and selected genes that were highly up-regulated in PE placentas. The gene expression RNA-seq datasets GSE114691 (*28*) and GSE148241 (*29*) were used. The GSE114691 dataset (Illumina HiSeq 2000 platform) contained 41 samples (20 Eo-PE samples and 21 controls). The GSE148241 series (Illumina HiSeq 2500 platform) contained 41 samples (9 Eo-PE samples and 32 controls). The SRA files were downloaded and converted to FASTQ files using a parallel-FASTQ dump. The expression level of each gene was quantified using the Kallisto (*55*). Data were summarized using the tximport package (ver. 1.18.0), and DEGs were identified using the Wald test in DESeq2 (ver. 1.36.0). DEGs were determined as genes with an adjusted *P* value < 0.05 and absolute log_2_FC > 1. We also searched for protein location (membrane protein or not) and expression levels in the placenta using the UniProt database (https://uniprot.org/) and Human Protein Atlas (https://proteinatlas.org/), respectively.

### Construction of plasmid DNA

Full-length *LIMCH1* was PCR-amplified with forward (5′-actgatgaattcaccATGGCTTGTCCCGCTCTCGG-3′) and reverse (5′-tacgatgcggccgctaTCACAATGTTGTAGGCTGCC-3′) primers from JAR cDNA. The amplification product was excised via NotI and EcoRI digestion and separated on a 1.0% agarose gel in 1 × tris-acetate-EDTA buffer. The band was extracted from the gel using a QIAquick Gel Extraction Kit (Qiagen, Hilden, Germany). The product was cloned into a FLAG-tagged pQCXIP retroviral vector (Clontech, Mountain View, CA, USA) predigested with NotI and EcoRI. The resultant plasmid was introduced into competent high DH5α (catalog no. DNA-903, TOYOBO Co. Ltd., Osaka, Japan), and transformants were selected on LB broth agar plate containing ampicillin (100 μg/ml). Positive clones were expanded by growth in LB broth medium containing ampicillin (100 μg/ml), and the plasmid was extracted using the QIAprep spin Miniprep Kit (Qiagen). The resulting pQCXIP-LIMCH1 clone was confirmed by sequencing. FLAG-tagged pQCXIP without the LIMCH1 insert was used as the transfection control.

### Transfection of plasmid DNA

Plasmid DNA of pQCXIP-FLAG or pQCXIP-FLAG-LIMCH1, vesicular stomatitis virus glycoprotein, and Gag/Pol were transfected into human embryonic kidney 293T cells using Lipofectamine 3000 (Thermo Fisher Scientific). Supernatant containing virus were mixed with Polybrene (2 μg/ml) and infected to JAR cells. Cellular clones expressing FLAG (Control) and FLAG-LIMCH1 (LIMCH1-OE) were selected with puromycin (1.0 μg/ml) for 2 weeks. We generated LIMCH1-OE BeWo cells by the same method as in the JAR except for the concentration of puromycin (2.5 μg/ml) (*56*).

### EV staining and cellular uptake

This method is illustrated in Fig. 4G. PBS was added to the 3 μg of JAR-EVs to reach a total volume of 200 μl. Next, 1 μl of CellMask Green plasma membrane stain (Invitrogen) was added and incubated for 30 min at 37°C and transferred to 1.5-ml microcentrifuge tube. The stained EVs were washed with PBS, ultracentrifuged at 110,000*g* for 70 min at 4°C using a TLA-55 rotor (Beckman Coulter). The pellet was washed again with PBS, ultracentrifuged under the same conditions, and resuspended in PBS to extract CellMask Green–labeled EVs. HUVECs were seeded on an ibiTreat μ-Slide eight-well plate (catalog no. 80826, Ibidi GmbH, Germany) at a density of 20,000 cells per well. The slides were stored in an incubator. After 12 hours, labeled EVs were injected into the culture medium. The cells were then incubated for 5 hours. The plasma membrane and nuclei were stained using CellMask Deep Red (Invitrogen) and Hoechst 33342 (Thermo Fisher Scientific), respectively. EV uptake was observed using a confocal fluorescence microscope (AX/AXR, Nikon, Japan). The images were analyzed using the NIS-Elements AR Analysis program.

### RNA extraction and RT-qPCR analysis

Total RNA was extracted from cultured cells using QIAzol and the miRNeasy Mini Kit (Qiagen), according to the manufacturer’s protocols. For RT-qPCR analysis, cDNA was generated from 500 ng of total RNA using ReverTraAce qPCR RT Master Mix (TOYOBO Co. Ltd). RT-qPCR was performed in technical triplicate with 2.5 ng of cDNA using TB Green Premix Ex Taq (TaKaRa Bio, Shiga, Japan) on a SYBR Green real-time PCR system. Data were collected and analyzed using Mx3000P (Agilent Technologies, USA) with MxPro software version 4.10. All mRNA quantification data from cultured cells were normalized to the expression of glyceraldehyde 3-phosphate dehydrogenase. All primer sequences are listed in table S1.

### RNA sequencing

HUVECs were seeded in 12-well plates at a density of 100,000 cells per well. After 6 hours, 30 μg of JAR-FLAG-sEVs (Control-EVs) or JAR-LIMCH1-sEVs (LIMCH1-EVs) were added. Twenty-four hours after EV injection, the cells were washed with PBS, and 500 μl of QIAzol was added to each well. RNA was extracted and the RNA concentration was measured using a Nanodrop 2000c spectrophotometer (Thermo Fisher Scientific). RNA library preparation and transcriptome sequencing were performed by Novogene Co. LTD (Beijing, China). Gene expression profiles were obtained as described above, and scaledTPM counts were used for further analyses. PCA and heatmap analyses were performed using DEGs identified by the Wald test in DESeq2 (ver. 1.36.0). DEGs were determined as genes with adjusted *P* value < 0.05 and absolute log_2_FC > 0.8. GSEA was performed to compare Control-EV–treated and LIMCH1-EV–treated HUVECs. GSEA is publicly available software developed by the Broad Institute (https://gsea-msigdb.org/gsea/index.jsp).

### Immunoblot analysis

Proteins (cell lysates and EVs) were denatured at 95°C in sample buffer solution with 3-Mercapto-1,2-propanediol (FUJIFILM Wako Pure Chemical Corporation) for 5 min and then loaded onto polyacrylamide gels for electrophoretic separation of proteins at 30 mA. The proteins were transferred onto polyvinylidene difluoride membranes. After blocking with Blocking One (Nacalai Tesque Inc., Japan) for 1 hour at 15° to 25°C, the membranes were incubated overnight at 4°C with the following primary antibodies: mouse monoclonal anti-CD9 (catalog no. CBL162; Merck; dilution 1:100), rabbit monoclonal anti-CD63 (catalog no. EXOAB-CD63A-1; System Biosciences LLC, CA, USA; dilution 1:1000), mouse monoclonal anti-CD81 (catalog no. sc-166029; Santa Cruz Biotechnology, TX, USA; dilution 1:100), rabbit monoclonal anti-PLAP (catalog no. ab133602; Abcam, Cambridge, UK; dilution 1:1000), and rabbit polyclonal anti-LIMCH1 (catalog no. ab96178; Abcam, Cambridge, UK; dilution 1:2000). Subsequently, the membranes were washed thrice for 5 min using tris-buffered saline with 0.1% Tween-20 and then incubated for 1 to 3 hours at 15° to 25°C with secondary horseradish peroxidase (HRP)–conjugated mouse anti-rabbit IgG (catalog no. NA934-1ML; Cytiva Life Sciences, USA; dilution 1:5000) or anti-mouse IgG (catalog no. NA931-1ML; Cytiva; dilution 1:2000) antibodies. To detect β-actin, a membrane was incubated for 1 hour with β-actin monoclonal antibody HRP-conjugated antibody (catalog no. 289-99361; FUJIFILM Wako Pure Chemical Corporation; dilution 1:5000). The membranes were imaged using an ImageQuant LAS 4010 (GE Healthcare, IL, USA).

### Immunohistochemical staining

Patient characteristics based on placental immunohistochemical analysis are shown in table S2. The Eo-PE cohort was the same as the serum sample collection cohort, whereas control placentas were collected from five women with iatrogenic preterm delivery because the placentas of the control cohort from which serum samples were collected were full-term placentas. The placental tissues were fixed in formalin and embedded in paraffin. Paraffin sections were deparaffinized and rehydrated, and antigens were retrieved using 10 mM sodium citrate buffer (pH 6.0) in a microwave oven. Endogenous peroxidase activity was blocked by incubation with 0.3% H_2_O_2_ in methanol for 30 min, and nonspecific IgG binding was blocked by treatment with 10% normal goat serum for 10 min. The sections were incubated at 4°C overnight with rabbit anti-LIMCH1 antibody (catalog no. ab96178; Abcam, Cambridge, UK; dilution 1:200) or rabbit anti-hCG beta antibody (catalog no. ab131170; Abcam, Cambridge, UK; dilution 1:100). After washing in PBS, the sections were incubated using a Histofine SAB-PO kit (Nichirei Bioscience Inc., Tokyo, Japan). Visualization was performed using 3,3′-diaminobenzidine and counterstaining with Meyer’s hematoxylin (FUJIFILM Wako Pure Chemical Corporation). The slides were scanned using a VS120 Slide Scanner (Olympus, Tokyo, Japan). Quantitative analysis was performed for LIMCH1 using HALO Image Analysis software (Indica Labs, USA). An *H* score was calculated to quantify immunohistochemistry scores, defined as follows: (1 × % cells 1+ staining intensity) + (2 × % cells 2+ staining intensity) + (3 × % cells 3+ staining intensity). Staining intensity was estimated using the following categories: 0 (negative staining), 1+ (weak staining), 2+ (moderate staining), or 3+ (strong staining) (*57*).

### Immunofluorescence analysis

HUVECs were seeded in eight-well chamber slides (catalog no. 5732-008, IWAKI, Japan) at a density of 50,000 cells per well. The slides were then stored in an incubator. Six hours after seeding, 6 μg of Control-EVs or LIMCH1-EVs were added. Twenty-four hours later, the cells were washed with PBS and fixed in 2% paraformaldehyde for 15 min. The cells were then permeabilized with 0.1% Triton X-100 for 10 min, blocked with 5% donkey serum for 30 min at 15° to 25°C, and then incubated with rabbit anti–ZO-1 antibody (catalog no. 13663S; Cell Signaling Technology, USA; dilution 1:100) overnight with 4°C, followed by incubation with secondary antibodies (Alexa Fluor 488–conjugated donkey anti-rabbit antibody; Invitrogen; dilution 1:500) and 4′,6-diamidino-2-phenylindole (DAPI, dilution 1:1000) for 1.5 hours at 15° to 25°C. The cells were washed three times, and the chamber was removed and mounted for examination under a microscope. ZO-1 expression was examined using confocal fluorescence microscopy (AX/AXR). The images were analyzed using the NIS-Elements AR Analysis program.

### Cell proliferation assay

HUVECs were seeded in 96-well plates at a density of 4000 cells per well. After 6 hours, 3 μg of Control-EVs or LIMCH1-EVs were added, and the confluences of the cells were measured every 6 hours until 24 hours using a live-cell imaging system, IncuCyte SX5 (Sartorius, Johnson Avenue, Bohemia, USA).

The effect of LIMCH1-OE on cell proliferation was examined using a CellTiter-Glo Luminescent Cell Viability Assay (Promega, USA). Control and LIMCH1-OE JAR cells were seeded into 96-well plates at a density of 2,000 cells per well. CellTiter-Glo reagent was added after 24, 48, 72, and 96 hours followed by 10-min incubation at 37°C and luminescence measured using Tecan Infinite F200 Pro microplate reader (Tecan, Switzerland).

### Tube formation assay

First, eight-well chamber slides (catalog no. 5732-008, IWAKI) were coated with 160 μl of growth factor-reduced Matrigel (10 mg/ml; catalog no. 356231, Corning, USA) and incubated in a 37°C incubator for 30 min. Then, 160 μl of medium was added and incubated for 30 min, followed by adding 160 μl of medium containing 40,000 cells and 9.6 μg of Control-EVs or LIMCH1-EVs and incubating for 16 hours. Images were obtained using BZ-X800 microscope (Keyence, Japan). The number of branching points was quantified using ImageJ software.

### Analysis of tight junction function by in vitro permeability assay and TEER

HUVECs were seeded on transwell inserts (0.4 μm pore, catalog no. 353095, Corning) in 24-well plates at a density of 50,000 cells per well. After 6 hours, 6 μg of Control-EVs or LIMCH1-EVs were added to the upper chamber. Twenty-four hours later, 40- or 70-kDa FITC-dextran (Sigma-Aldrich, USA) at a final concentration of 1 mg/ml was added to the upper chamber. At the indicated time points (0 min indicates the time before FITC-dextran injection), 50 μl of samples were taken from the lower chamber and replaced with the same volume of growth medium. The fluorescence content of the samples was measured at an excitation wavelength of 485 nm and an emission wavelength of 535 nm using a Tecan Infinite F200 Pro microplate reader (Tecan).

In addition, we used TEER measurements, a technique that quantitatively evaluates the barrier function of endothelial monolayers by measuring electrical resistance. The experiments were conducted according to the same procedure as described above, except that ad-MED Vitrigel2 inserts (catalog no. 08364-96, Kanto Chemical, Tokyo, Japan) were used. TEER values were measured with an electrical resistance measurement system (Kanto chemical, Tokyo, Japan).

### In vitro hypoxia treatment

JAR cells were seeded in six-well plates at densities of 150,000, 75,000, and 37,500 cells per well for 24, 48, and 72 hours of hypoxia treatment, respectively, and incubated under normoxic conditions (20% O_2_). After 12 hours, plates assigned to the normoxic group were maintained in the same incubator, while those assigned to the hypoxic group were transferred to a hypoxic incubator (1% O_2_). Total RNA was extracted 24, 48, and 72 hours after the initiation of hypoxic treatment.

### Processing and annotation of reference scRNA-seq data

We analyzed scRNA-seq data from GSE173193 to construct a cell-type reference for the deconvolution of spatial transcriptomics data. From this dataset, we used two PE cases (GSM5261699 and GSM5261700) and two control cases (GSM5261695 and GSM5261696). Using Seurat (v5.0.0) according to its official vignette (https://satijalab.org/seurat/), we performed quality control, normalization with the “NormalizeData” function, and data scaling with the “ScaleData” function. Dimensionality reduction was conducted using PCA, followed by integration with Harmony to correct for batch effects. Clustering was performed with the “FindClusters” function at a resolution of 0.6, and cell-type annotation was carried out using established marker genes. When we examined the expression of LIMCH1, we observed differential expression levels across SCT clusters. SCT clusters with elevated LIMCH1 expression were therefore designated as LIMCH1-positive SCT [SCT LIMCH1(+)].

### Spatial transcriptome analyses

For spatial transcriptomics, we used the Visium CytAssist Spatial Gene Expression for formalin-fixed, paraffin-embedded (FFPE) (10x Genomics, USA). In this analysis, the whole RNA transcriptome of cells in each spot of the specialized slides was obtained from FFPE tissue sections. Each spot contained approximately 10 to 20 cells, and the RNA transcriptome of these cells revealed the features of RNA expression in each spot. Among the placentas immunohistochemically stained for LIMCH1, a PE placenta with high LIMCH1 expression and a control placenta with low LIMCH1 expression were selected. The maternal side of the placenta (5 μm) was mounted on each captured area. H&E staining, RNA library preparation, and transcriptome sequencing were performed by CyberomiX (Kyoto, Japan). The sections were stained with H&E, imaged, and decoverslipped, followed by H&E destaining and decross-linking. Glass slides with tissue sections were processed using a Visium CytAssist instrument to transfer the analytes to a Visium CytAssist Spatial Gene Expression slide with an 11 mm by 11 mm capture area. The probe extension and library construction steps followed the standard Visium for the FFPE workflow. Libraries were sequenced using a DNBSEQ-G400 sequencer (BGI) [read 1: 100 base pair (bp); read 2: 100 bp].

The Visium sequencing data were processed using Space Ranger (2.0.1, 10x Genomics). The output count matrix and image data were normalized, quality controlled, and dimension reduced using the R package Seurat (v5.0.0). Spots with an nCount of 0 were removed, and normalization was performed using the SCTransform function in Seurat. Reduction and clustering were performed according to its vignettes.

### Deconvolution

We used the CARD R package (*58*) to estimate the cell-type composition of each spot in the Visium spatial transcriptome data, using the processed scRNA-seq data as a reference. Following the CARD workflow (https://github.com/YMa-lab/CARD), a CARD object was created with the “createCARDObject” function by incorporating the count matrix and spatial location information from the processed Visium Seurat object, the scRNA-seq reference data, and the corresponding cell-type labels. Cell type-deconvolution was then performed using the “CARD_deconvolution” function, and the estimated cell-type proportions were visualized. The “CARD.visualize.pie” function was used to generate pie charts showing the relative proportions of all deconvoluted cell types. From all spots in the PE sample, those ranking in the top 10% for the presence of SCT LIMCH1(+) were designated as “SCT LIMCH1-high” spots, and those ranking in the top 10% for the presence of SCT were designated as “SCT LIMCH1-low” spots. Spots falling into both categories were excluded from further analysis. DEGs between these two groups were identified using the “FindMarkers” function with test.use = “MAST.” Genes with absolute log_2_FC > 1 were extracted, and the pathway analysis was subsequently performed using Ingenuity Pathway Analysis.

### ExoView assay

The expression of LIMCH1 in PE serum sEVs was examined using ExoView Tetraspanin Kits (NanoView Biosciences, MA, USA). EV samples were diluted 500-fold with incubation solution, and then an 80-μl EV sample was incubated for 16 hours at 15° to 25°C on ExoView Tetraspanin chips and placed in a sealed 24-well plate. The chips contained spots labeled with anti-CD9, anti-CD63, or anti-CD81 as EV markers, or mouse IgG1κ isotype antibody used as a control for nonspecific EV binding. The chips were washed in solution A for 3 min, permeabilized in PBS containing 0.02% Triton X-100 for 3 min at 15° to 25°C, and then washed three times in 1 ml of solution A for 3 min under gentle shaking, followed by incubation for 1 hour at 15° to 25°C under shaking at 500 rpm with the mixture of anti-LIMCH1 fluorescence antibodies, containing NBP2-97832AF594 (Novus Biologicals, USA), and AF488-labeled ab96178 (abcam), all similarly diluted (1:300) in blocking solution. Anti-LIMCH1 antibody, ab96178, was labeled using the antibody labeling kit Alexa Fluor 488 (catalog no. A20181, Thermo Fisher Scientific). The chips were then washed once in solution A and four times in solution B, rinsed with Milli-Q water, and dried. The chips were imaged with an ExoView R100 imager (Nanview Biosciences) using ExoScan version 2.5.5 acquisition software. The acquired images were analyzed using ExoView Analyzer version 3.1 software. Since the baseline signal was subtracted, some negative values were observed.

### Nanoflow cytometry

To examine the expression of LIMCH1 and PLAP in serum sEVs from control and PE pregnant women, as well as placental sEVs, we used NanoFCM (Flow NanoAnalyzer, NanoFCM Inc.). Placental EVs were isolated according to previously published method (*59*), and culture medium used was Iscove’s modified Dulbecco’s medium (Thermo Fisher Scientific Inc., USA) supplemented with 2% exosome-depleted FBS (EXO-FBS-50A-1, SBI, Japan). LIMCH1 was stained with the C30106-AF647 antibody (Signalway Antibody, USA) at a working concentration of 2 μg/ml, and PLAP was stained with the sc47691-AF488 antibody (Santa Cruz Biotechnology, TX, USA) at a working concentration of 2 μg/ml. Data were analyzed using NanoFCM Profession software (version 2.0).

### In vivo studies

The experimental procedures in this study were approved by the Animal Experiment Committee of Nagoya University Graduate School of Medicine (approval number: M240037-001), complied with the ARRIVE guidelines, and were performed in accordance with the National Institutes of Health Guide for the Care and Use of Laboratory Animals. Sample sizes were determined on the basis of prior experience with similar in vivo experiments and feasibility considerations. No predefined inclusion or exclusion criteria were applied. Nonpregnant female ICR mice aged 7 to 8 weeks and pregnant ICR mice aged 8 to 12 weeks (The Jackson Laboratory Japan Inc., Yokohama, Japan) were used in the experiments. The method used for in vivo vascular permeability assay is illustrated in Fig. 6A. The mice were administered 100 μl of PBS containing 10 μg of Control-EVs (*n* = 6) or LIMCH1-EVs (*n* = 6) retro-orbitally. Twenty-four hours later, 100 μl of 2% Evans blue dye solution (Sigma-Aldrich, USA), which can estimate vascular leakage, was injected retro-orbitally (*60*). In pregnant mice, blood pressure was measured on gestational day 13 (GD13), followed by intravenous injection of EVs (15 μg/100 μl per mouse). On GD16, a second intravenous injection of EVs (15 μg/100 μl per mouse) was administered. On GD17, blood pressure measurement, urine collection, and fetal assessments were performed, or Evans blue was administered. After 1 hour, the mice were euthanized and perfused PBS via the heart, and lung, and brain tissues were collected. The lung and brain weights were measured and placed in 10% neutral-buffered formalin at 60°C for 72 hours to extract Evans blue dye. The samples were centrifuged at 5000*g* for 20 min, and the supernatant was collected to quantify the concentration of Evans blue dye by measuring the absorbance at 620 nm using a Viento 808 IU absorbance reader (BioTek, Winooski, VT, USA). The Evans blue dye concentration was calculated using a standard curve. Data were converted into micrograms of extravasated dye per gram of tissue. Blood pressure was noninvasively measured using an automatic blood pressure analyzer with a tail-cuff device (BP-98AL; Softron, Tokyo, Japan). The mean of three consecutive readings was recorded for each mouse. Urine samples were collected on GD17. Urinary creatinine and albumin levels were measured using Creatinine Companion and Albuwell M kits (Exocell Inc., Pholadelphia, PA, USA), respectively.

For in vivo EV uptake experiment, the mice were injected with 100 μl of CellMask Green–labeled PBS (*n* = 1) or CellMask Green–labeled 10 μg of JAR EVs (*n* = 3). To generate CellMask green-labeled PBS, 1 μl of CellMask Green plasma membrane stain (Invitrogen) was added to 200 μl of PBS and incubated for 30 min at 37°C and transferred to a 1.5-ml microcentrifuge tube. The stained PBS was washed with PBS, ultracentrifuged twice at 110,000*g* for 70 min at 4°C using a TLA-55 rotor (Beckman Coulter), and resuspended in PBS. CellMask Green–labeled EVs were generated using the method described for “EV staining and cellular uptake.” Twenty-four hours after the injection, the mice were euthanized and perfused with 4% paraformaldehyde. Lung, brain, and kidney tissues were dissected, fixed in 2% paraformaldehyde at 4°C for 30 min, washed three times in cold PBS, and incubated in 20% sucrose at 4°C overnight. The tissues were then embedded in OCT compound (Sakura Finetek, Japan), cut into 10-μm sections, and stained with DAPI (dilution 1:1000). The slides were observed under a confocal fluorescence microscope (AX/AXR).

### Statistical analysis

RStudio (RStudio Inc.) and R software (ver. 4.0.3) or SPSS v29 (IBM, Armonk, NY, USA) were used. Unless otherwise stated, the data are presented as the means ± SD and statistical significance was determined by a Student’s *t* test. Statistical *P* value < 0.05 was considered as statistically significant.
