# Supplementary material for: LIMCH1-enriched extracellular vesicles promote vascular permeability in early-onset preeclampsia
Source: Sci Adv. 2026 Jan 28;12(5):eaeb8806. doi: 10.1126/sciadv.aeb8806 (PMC12851021; doi:10.1126/sciadv.aeb8806)
Supplement: Supplementary file 1 — Figs. S1 to S10 Tables S1 and S2 Legend for table S3 [file sciadv.aeb8806_sm.pdf]

Science Advances
AAAS

Supplementary Materials for

LIMCH1-enriched extracellular vesicles promote vascular permeability in early-onset preeclampsia

Seiko Matsuo *et al.*

Corresponding author: Akira Yokoi, ayokoi@med.nagoya-u.ac.jp

Sci. Adv. **12**, eaeb8806 (2026)
DOI: 10.1126/sciadv.aeb8806

The PDF file includes:

Figs. S1 to S10
Tables S1 and S2
Legend for table S3

Other Supplementary Material for this manuscript includes the following:

Table S3

Fig. S1.

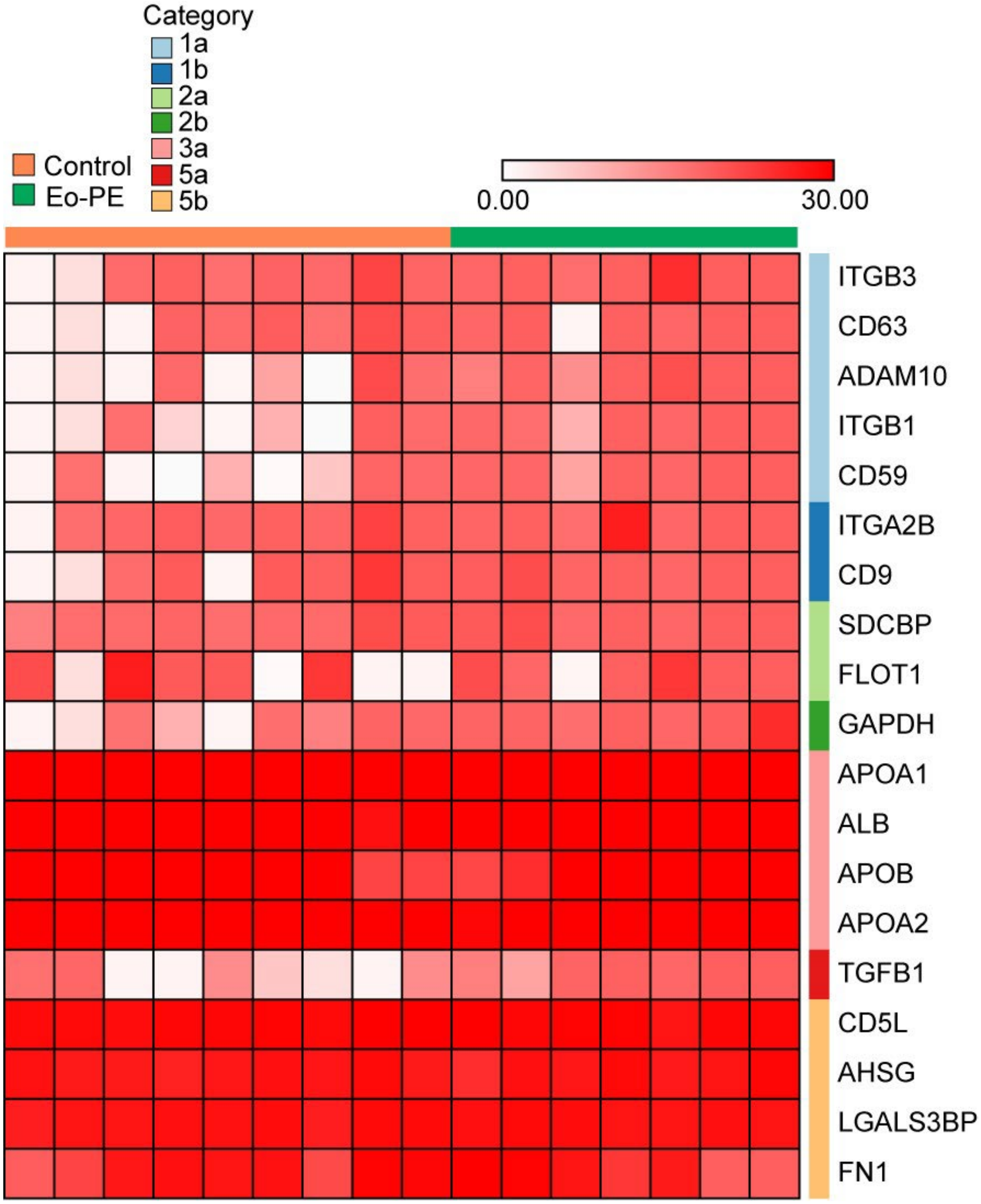

Supplementary Fig. S1. Expression profiles of EV-associated proteins of control and Eo-PE serum sEVs

Quantitative imaging of the mass spectrometry data. According to the protein content-based EV characterization from MISEV2018, EV-associated proteins were categorized into 1a to 5b.

Fig. S2.

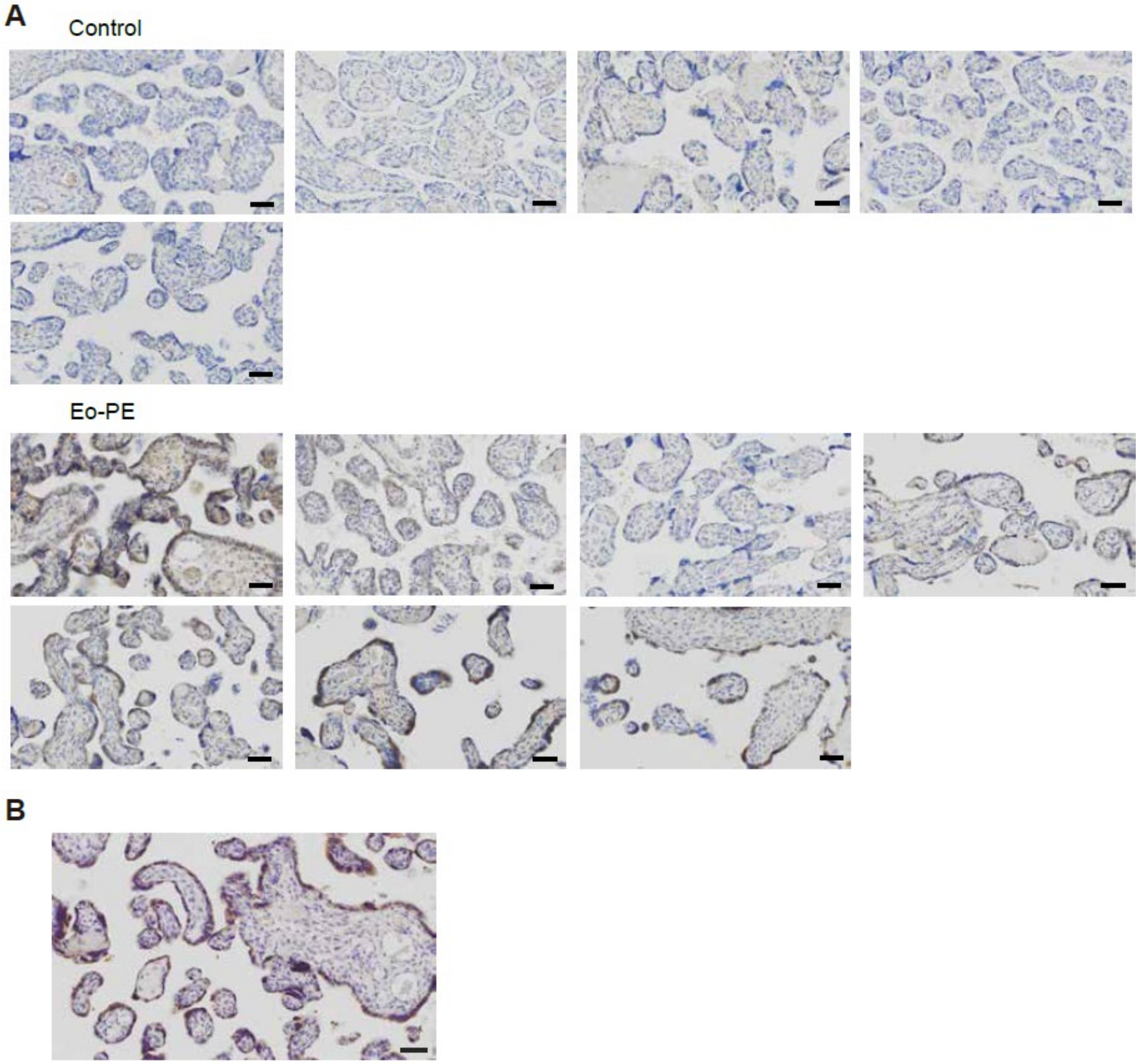

Supplementary Fig. S2. LIMCH1 is predominantly expressed in syncytiotrophoblasts

(A) Images of immunohistochemical staining for LIMCH1 in control and Eo-PE placentas. Scale bar represents 50 μm. One of the Eo-PE images shown in this figure is the same image presented in Fig. 2E.

(B) Images of immunohistochemical staining for hCG in the Eo-PE placenta. Scale bar represents 50 μm.

Fig. S3.

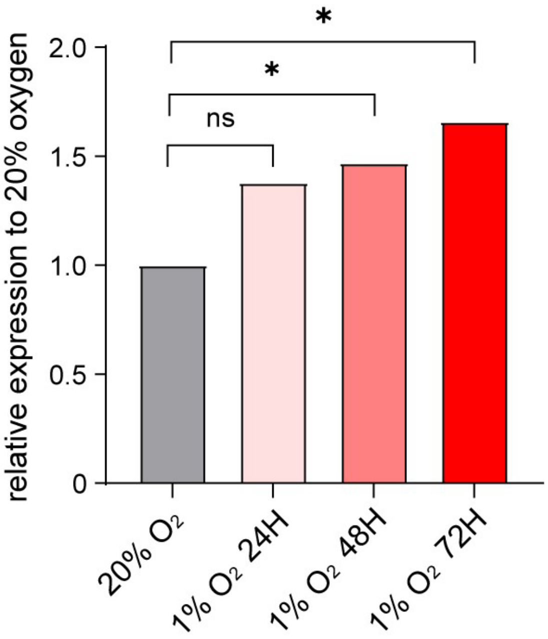

Supplementary Fig. S3. LIMCH1 expression is upregulated under hypoxic conditions

LIMCH1 expression was measured by real-time quantitative PCR, and GAPDH was used as internal control. *$p < 0.05$, ns, no significance, Student's *t*-test. Data are representative of at least three independent experiments.

Fig. S4.

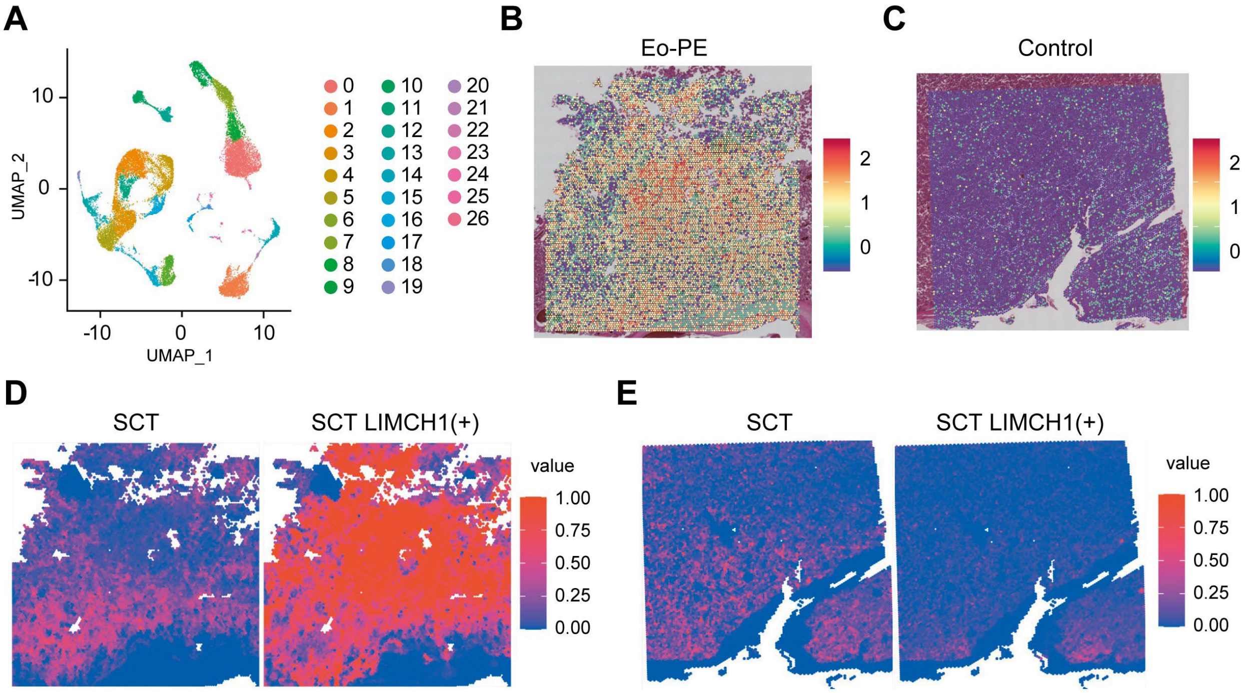

Supplementary Fig. S4. Supplementary results of the spatial transcriptomic analysis

(A) Uniform manifold approximation and projection (UMAP) plot of unsupervised clustering of single-cell RNA-sequencing dataset GSE173193.

(B-C) Spatial feature plots of LIMCH1 in the control and Eo-PE placentas.

(D) Cell type proportion of SCT and SCT LIMCH1(+) of the Eo-PE placenta.

(E) Cell type proportion of SCT and SCT LIMCH1(+) of the control placenta.

The spatial images here correspond to truncated regions of the same Eo-PE and control placental tissue sections shown in Fig. 3 and were generated using different analytical processing pipelines.

Fig. S5.

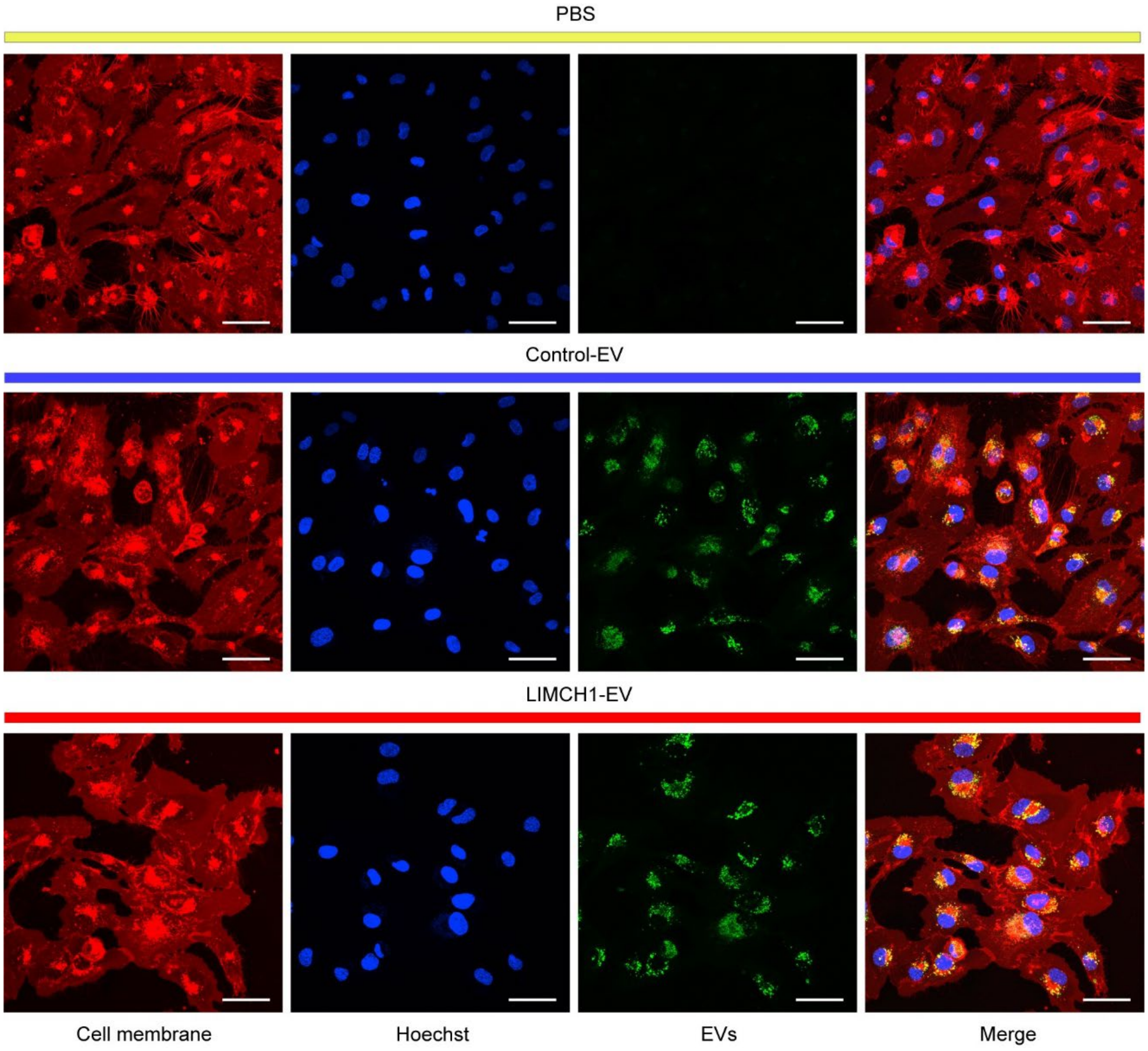

Supplementary Fig. S5. Uptake of EVs in HUVECs

Representative images using confocal microscopy. EVs were labelled with CellMask^{TM} Green, and the cell membrane and nuclei were stained with CellMask^{TM} Deep Red and Hoechst, respectively. Scale bar represents 50 µm. Portions of these images are shown as Fig. 4H.

Fig. S6.

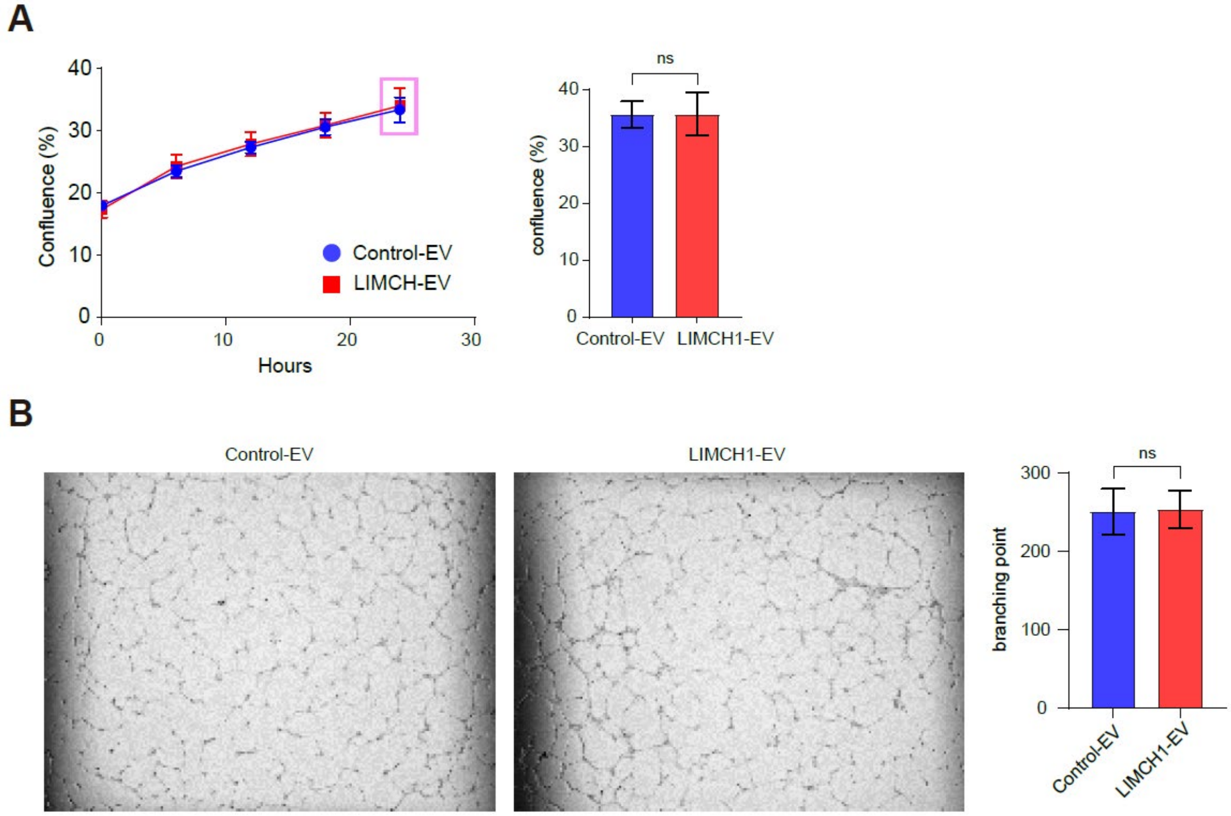

Supplementary Fig. S6. LIMCH1-EVs do not alter proliferation or tube formation ability

(A) Quantitative results of cell proliferation assay of Control-EV- and LIMCH1-EV-treated HUVECs. Confluence (%) after 24 h was compared. Data are shown as mean ± standard deviation. NS, no significance, Student’s *t*-test.

(B) Representative images using BZ-X800 microscopy. The number of branching points was quantified using ImageJ software. The graph on the right shows quantitative results. Data are shown as mean ± standard deviation. NS, no significance, Student’s *t*-test.

Data are representative of at least three independent experiments [(A) and (B)].

Fig. S7.

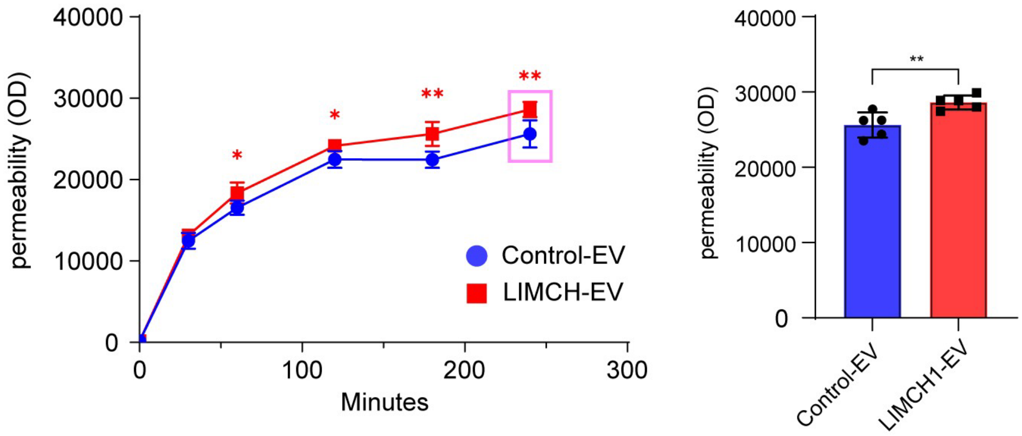

Supplementary Fig. S7. HUVEC *in vitro* permeability assay using EVs released from LIMCH1-overexpressing JAR cells generated from another clone

Quantitative results of *in vitro* permeability assay. The transmitted FITC was measured as the optical density (OD), and the OD after 4 h was compared. Data are shown as mean ± standard deviation. *$p < 0.05$, **$p < 0.01$, Student's *t*-test. Data are representative of at least three independent experiments.

Fig. S8.

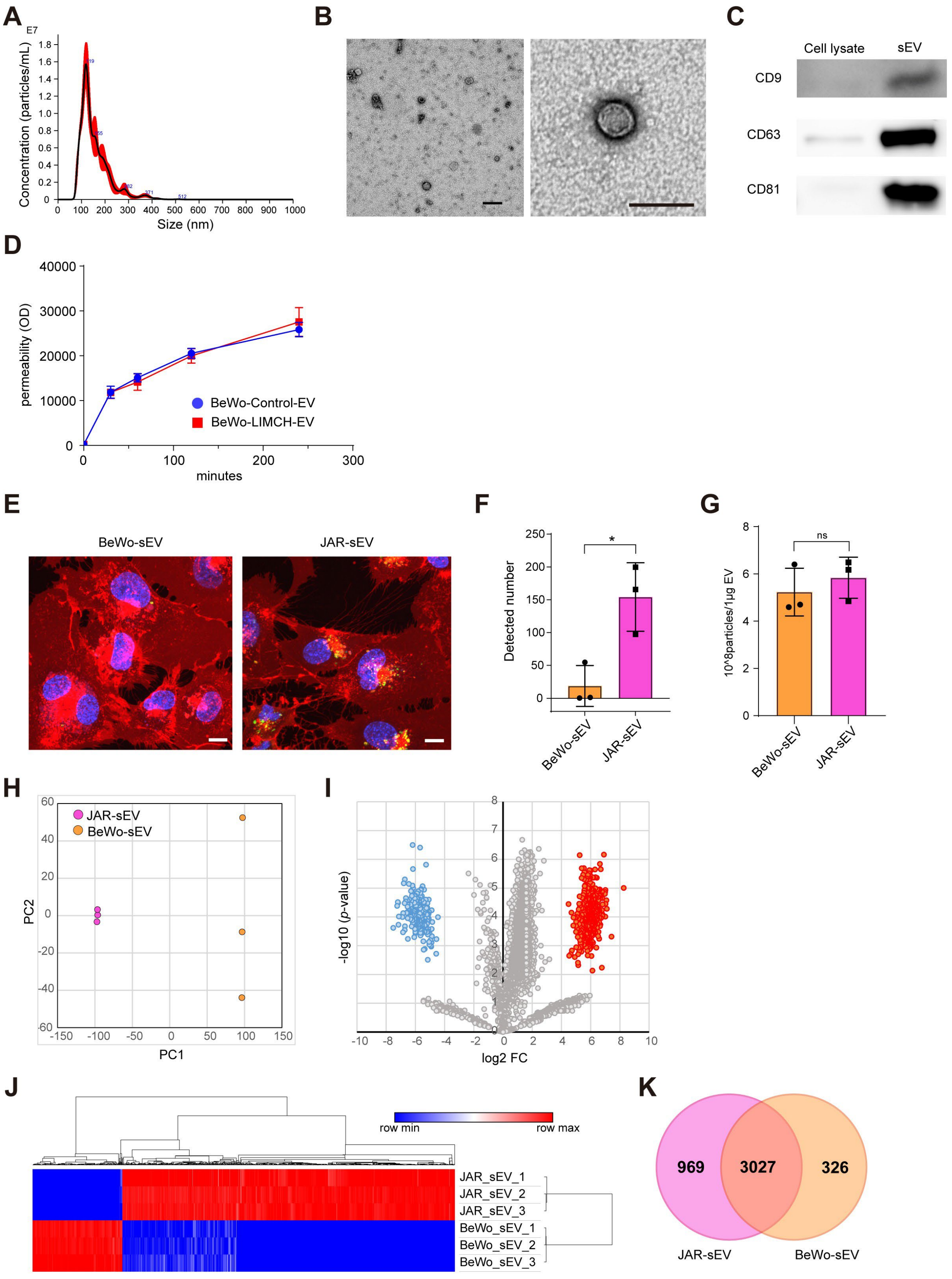
A
E7
Concentration (particles/mL)
Size (nm)
B
C
Cell lysate
sEV
CD9
CD63
CD81
D
permeability (OD)
minutes
BeWo-Control-EV
BeWo-LIMCH-EV
E
BeWo-sEV
JAR-sEV
F
Detected number
*
BeWo-sEV
JAR-sEV
G
10^8particles/1μg EV
ns
BeWo-sEV
JAR-sEV
H
JAR-sEV
BeWo-sEV
PC2
PC1
I
-log10 (p-value)
log2 FC
J
row min
row max
JAR_sEV_1
JAR_sEV_2
JAR_sEV_3
BeWo_sEV_1
BeWo_sEV_2
BeWo_sEV_3
K
969
3027
326
JAR-sEV
BeWo-sEV

Supplementary Fig. S8. BeWo-LIMCH1-EVs do not increase transendothelial permeability

(A) Size distribution of BeWo small extracellular vesicles (sEVs) was observed using nanoparticle tracking analysis.

(B) Representative transmission electron microscopy images at low- and high-power fields of BeWo-sEVs. Scale bar of the left and right image represents 200 and 100 nm, respectively.

(C) Tetraspanin (CD9, CD63, and CD81) marker expression of cell lysates and sEVs of BeWo cells.

(D) Quantitative results of *in vitro* permeability assay. The transmitted FITC was measured as optical density (OD). Data are shown as mean ± standard deviation.

(E) Representative images using confocal microscopy. EVs were labelled with CellMask^{TM} Green and cell membrane and nuclei were stained with CellMask^{TM} Deep Red (red) and Hoechst (blue), respectively. Three micrograms of BeWo-sEVs or JAR-EVs were administered. Scale bar represents 10 μm.

(F) Quantitative result of BeWo-sEV or JAR-sEV uptake by HUVECs using NIS-Elements AR Analysis program. Data are shown as mean ± standard deviation. *$p < 0.05$, Student's *t*-test.

(G) Quantitative result of Nanoparticle Tracking Analysis for BeWo-sEVs and JAR-sEVs. Data are shown as mean ± standard deviation. NS, no significance, Student's *t*-test.

(H) Principal component analysis mapping for protein profiles of BeWo-sEVs and JAR-sEVs.

(I) Volcano plot showing differentially expressed genes between BeWo-sEVs and JAR-sEVs.

(J) Heatmap showing protein profiles of BeWo-sEVs and JAR-sEVs.

(K) Venn diagram showing the number of proteins detected in BeWo-sEVs and JAR-sEVs.

Data are representative of at least three independent experiments [(D) to (G)].

Fig. S9.

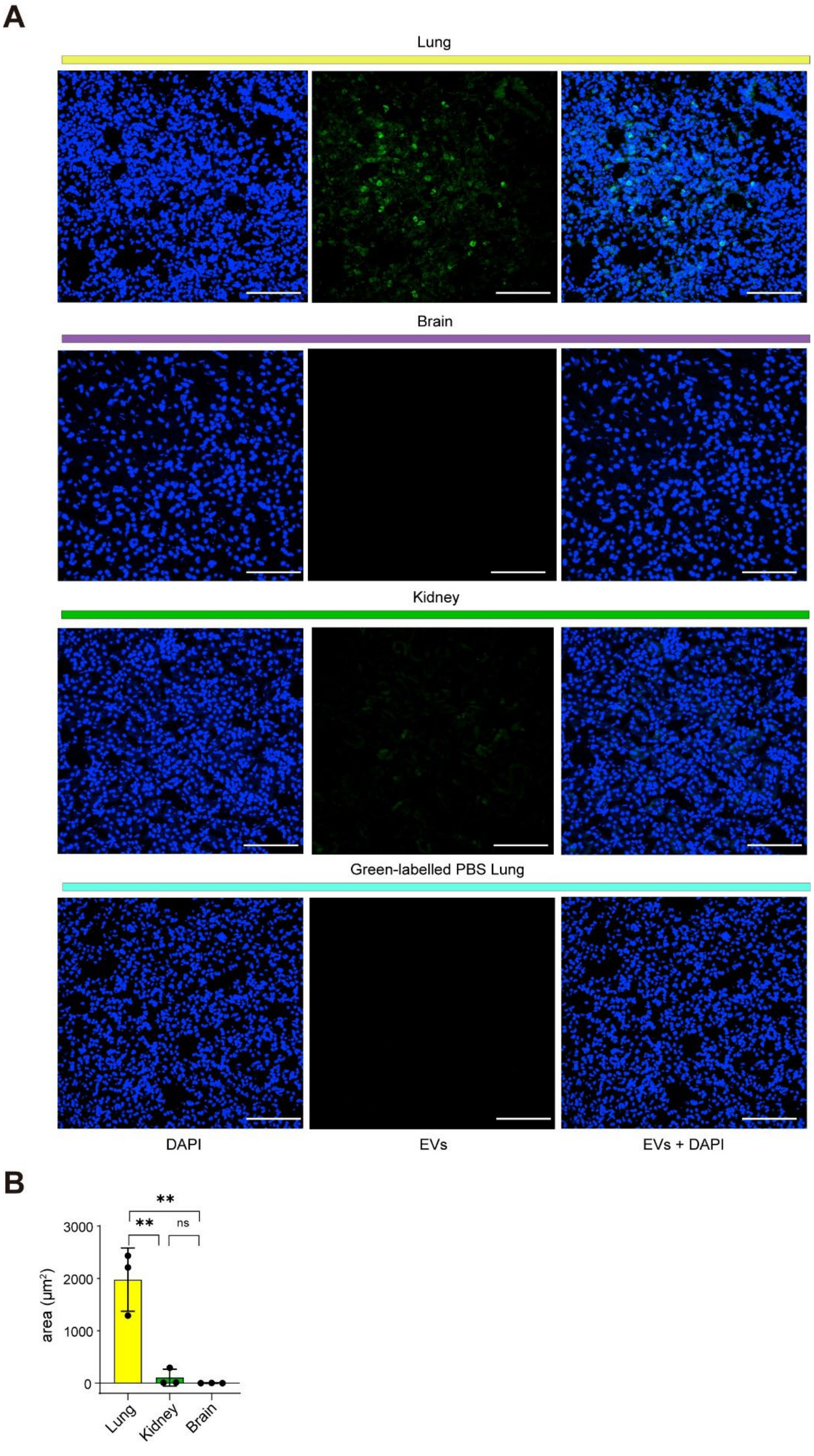
A
Lung
Brain
Kidney
Green-labelled PBS Lung
DAPI
EVs
EVs + DAPI
B
area (μm²)
3000
2000
1000
0
**
**
ns
Lung
Kidney
Brain

Supplementary Fig. S9. The brain and kidney show less EV uptake than the lung

(A) Representative images using confocal microscopy. EVs were labelled with CellMask^{TM} Green and ICR mice were administered CellMask^{TM} Green-labelled PBS (n = 1) or CellMask^{TM} Green-labelled 10 μg JAR EVs (n = 3). The nuclei were counterstained blue with DAPI. Scale bar represents 50 μm. Portions of these images are shown as Fig. 6E.

(B) Graph shows a quantitative result of NIS-elements AR Analysis program. Data are shown as mean ± standard deviation. $^{**}p < 0.01$; ns, no significance, Student's *t*-test.

Fig. S10.

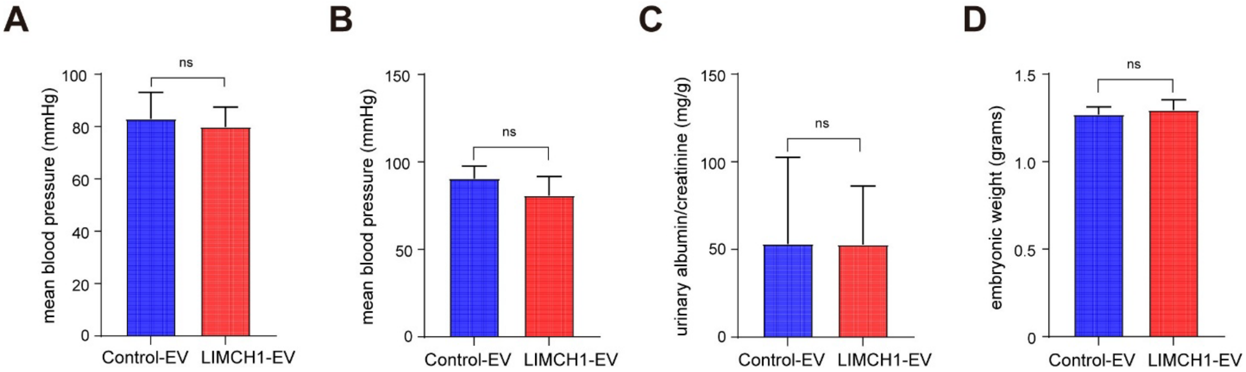

Supplementary Fig. S10. No significant differences in blood pressure, urinary protein levels, or embryonic weight following LIMCH1-EV administration

(A) Graphs showing the mean blood pressure results on GD13. Data are shown as mean ± standard deviation. NS, no significance, Student's *t*-test.

(B) Graphs showing the mean blood pressure results on GD17. Data are shown as mean ± standard deviation. NS, no significance, Student's *t*-test.

(C) Graphs showing a quantitative result of urinary albumin/creatinine level on GD17. Data are shown as mean ± standard deviation. NS, no significance, Student's *t*-test.

(D) Graphs showing the embryonic weight on GD17. Data are shown as mean ± standard deviation. NS, no significance, Student's *t*-test.

Table S1. Primer Sequences.

Gene	Forward	Reverse
GAPDH	CCAGGGCTGCTTTTAACTC	GCTCCCCCCTGCAAATGA
LIMCH1	ACAAAATGCCTGAAGCCAAC	CTGCGTTCTTCCTCTTCCAC
TJP1	GCTAAGAGCACAGCAATGGA	GCATGTTCAACGTTATCCAT
TJP2	CAAAAGAGGATTTGGAATTG	GAGCACATCAGAAATGACAA
RHOC	CGGGAGGTGTTTGAGATGG	CTGGGGTTGTAGGGGGATAAT
GJA5	CCGTGGTAGGCAAGGTCTG	ATCACACCGGAAATCAGCCTG
JUP	TCGCCATCTTCAAGTCGGG	AGGGGCACCATCTTTTGCAG
MTDH	GATGATGAATGGTCTGGGTTAAA	GACCTTTTGATCATCAGGAATTG
CTNNB1	AAAGCGGCTGTTAGTCACTGG	CGAGTCATTGCATACTGTCCAT
HGF	GTAAATGGGATTCCAACACGAACAA	TGTCGTGCAGTAAGAACCCAACTC
PTK2B	GGGAGGTCTATGAAGGTGTCTA	CTTCTCCTTGTTGTCCAGAGTG
AKT1	GTCATCGAACGCACCTTCCAT	AGCTTCAGGTACTCAAACTCGT
EPAS1	ATAAGTTCACCCAAAACCCCAT	GGCAGCAGGTAGGACTCAAAT
CLDN5	CTCTGCTGGTTCGCCAACAT	CAGCTCGTACTTCTGCGACA

Table S2. Patient characteristics based on placental immunohistochemical analysis.

	Control (n = 5)	Eo-PE (n = 7)	*p*-value
Maternal age (years)	34.8 ± 4.7	38.6 ± 4.0	0.16
BMI (kg/m2)	21.9 ± 2.0	20.9 ± 0.5	0.31
Primipara	1/5 (20.0%)	5/7 (71.4%)	0.12
Gestational age at delivery (weeks)	29.6 ± 1.4	28.0 ± 3.0	0.26
Birth weight (g)	1,342 ± 260	811 ± 329	0.01
Male	2/5 (40.0%)	6/7 (85.7%)	0.15

Data are shown as mean ± standard deviation or n (%). Statistical analyses were performed using the chi-squared test or Fisher's exact test for categorical variables and Student's *t*-test or Mann–Whitney U-test for continuous variables according to normal or non-normal distributions.

Eo-PE, early-onset preeclampsia; BMI, body mass index.

Table S3. Serum EV proteomic data from non-pregnant women.

Large and small EVs were isolated from the serum of three non-pregnant women and subjected to proteomic analysis.
